# Probiotic‐Based Materials as Living Therapeutics

**DOI:** 10.1002/adma.202508500

**Published:** 2025-09-25

**Authors:** Laura Sabio, Graham J. Day, Manuel Salmeron‐Sanchez

**Affiliations:** ^1^ Centre for the Cellular Microenvironment (CeMi) University of Glasgow University Avenue Glasgow G12 8QQ United Kingdom; ^2^ Institute for Bioengineering of Catalonia (IBEC) The Barcelona Institute for Science and Technology (BIST) Carrer de Baldiri Reixac Barcelona 08028 Spain; ^3^ Institució Catalana de Recerca i Estudis Avançats (ICREA) Passeig de Lluís Companys, 23 Barcelona 08010 Spain

**Keywords:** engineered living materials, probiotics, therapeutics

## Abstract

The growing demand for safer, more targeted therapeutics requires the development of advanced biomaterials. Among these, Engineered Living Materials (ELMs)—which integrate synthetic biology with material science—are emerging as promising platforms for biomedical applications. This review focuses on a subclass of ELMs based on genetically engineered probiotics combined with matrices, that are termed Probiotic Living Materials (PLMs) to differentiate them from Living Biotherapeutic Products (LBPs). Recent studies highlight PLM's potential in addressing different health conditions, offering targeted and dynamic therapies. However, PLMs face multiple challenges to be implemented in clinics, including a lack of robust genetic toolkits for probiotic engineering, concerns about biosafety (e.g., horizontal gene transfer or non‐desirable biological activity), difficulties in translating preclinical results to humans, and the absence of clear regulatory guidance for clinical use. This review first explores the fundamental features of ELMs, then provides an overview of probiotics, followed by recent advances in the design of engineered PLMs for biomedical applications, particularly in biosensing development, infection treatment, bone repair, wound healing, vaginal imbalances, gut‐related conditions, and cancer therapy. Finally, biosafety issues and current gaps in regulatory frameworks to ensure safe and effective use of PLMs, with a particular focus on vulnerable populations, are discussed.

## Graphical abstract

Recent advances in Engineered Living Materials are highlighted, integrating synthetic biology and advanced materials, with a focus on probiotic‐based therapeutics. Probiotic Living Materials hold great potential for biosensing, infection treatment, osteogenesis, wound healing, vaginal and gastrointestinal disorders, and cancer therapy. breakthroughs in the field is critically reviewed, including challenges for clinical translation.

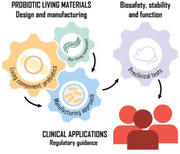

## 1 Introduction

In nature, living systems have evolved over millions of years to sense and adapt to external stimuli, turning into responsive and efficient biosynthetic factories. Some of the most impressive properties of living systems include their capacity for growth and self‐renewal, adaptation to the environment, ability to metabolize and synthesize an immense variety of organic and inorganic compounds, and their longevity and sustainability. Taking inspiration from them, new tools and lab‐grown living systems have been developed, attempting to simulate the “smartness” of these natural entities. Insights into their behavior, combined with the integration of synthetic biology and materials science has given rise to the emerging field of Engineered Living Materials (ELMs). This field has experienced significant breakthroughs in recent years, paving the way for the development of smart responsive materials with extensive applications in diverse fields such as bioproduction,^[^ ^1^ ^]^ construction,^[^ ^2^ ^]^ and biomedicine,^[^ ^3^ ^]^ among others.

This new category of biomaterial is based on the combination of a living and a non‐living component to obtain new, improved or similar functionalities compared to traditional (bio)materials that are not achievable by using one of the components individually. These functionalities are mainly focused in obtaining smart materials with higher complexity in terms of structure, organization and responsiveness, to obtain living materials that: i) can repair, grow and remodel themselves after damage, ii) can sense environmental cues and respond dynamically in real time, iii) can evolve and adapt to external changes, iv) are sustainable and renewable since they are usually made with or by living cells, and v) can deliver targeted treatments more precisely and minimize side effects.^[^ ^4^ ^]^

However, there is still a lack of consensus in the definition of ELM. The meaning of “engineered” refers to something that is intentionally designed and manipulated to meet specific performance requirements for an intended purpose. When it comes to living materials, such modifications are targeted at the living component. In this regard, a living material can be “chemically engineered” when living cells are encapsulated in a hydrogel, their surface is chemically coated or conjugated, or the cell itself is used for intracellular encapsulation; or “biologically engineered”, which refers to genetic engineering, membrane cloaking, and surface modification.^[^ ^5^ ^]^ On the other hand, in 2023, the category of “physical engineering” was included, which encompasses any electrostatic adsorption of nanoparticles, layer‐by‐layer encapsulation, and coating, leaving the concept of “chemical engineering” for purely chemical modifications on the cell surface.^[^ ^6^ ^]^ Despite all the efforts to categorize and define what an ELM is, there is a clear overlap with other fields, such as tissue engineering and bacterial encapsulation, blurring the boundaries between this emerging concept and previous ones.

The European Innovation Council published in 2021 the “EIC Pathfinder challenge: Engineered living materials,” where an ELM is defined as a material composed, either entirely or partly, of living cells.^[^ ^7^ ^]^ In this call, a distinction was made between biological ELMs (bELMs), entirely composed of living cells built via bottom‐up processes, and hybrid living materials (HLMs) or hybrid ELMs (hELMs), composed of living cells integrated within scaffolds via top‐down processes. Nowadays, the development of ELMs closely aligns to the advancement of genetic engineering tools, enabling the design of more sophisticated biomaterials with unique properties. Although the living cellular component can belong to any kingdom, the use of microbes is especially relevant for ELM development. Among them, probiotics arise as a more suitable candidate for ELM‐based therapies as a safer and commercially approved option.

This review aims to provide an overview of ELMs in general before focusing on the recent progress in the use of probiotics and the design of novel Probiotic‐based Living Materials (PLMs). Next sections focus on the main manufacturing techniques of ELMs, their composition and properties, followed by an introduction to probiotics and an extended discussion on genetic engineering of them, the design of PLMs with potential therapeutic applications, the challenges that this technology is currently facing regarding biosafety and regulatory frameworks, and future prospects.

### 1.1 ELM Design and Applications

**Figure 1:**
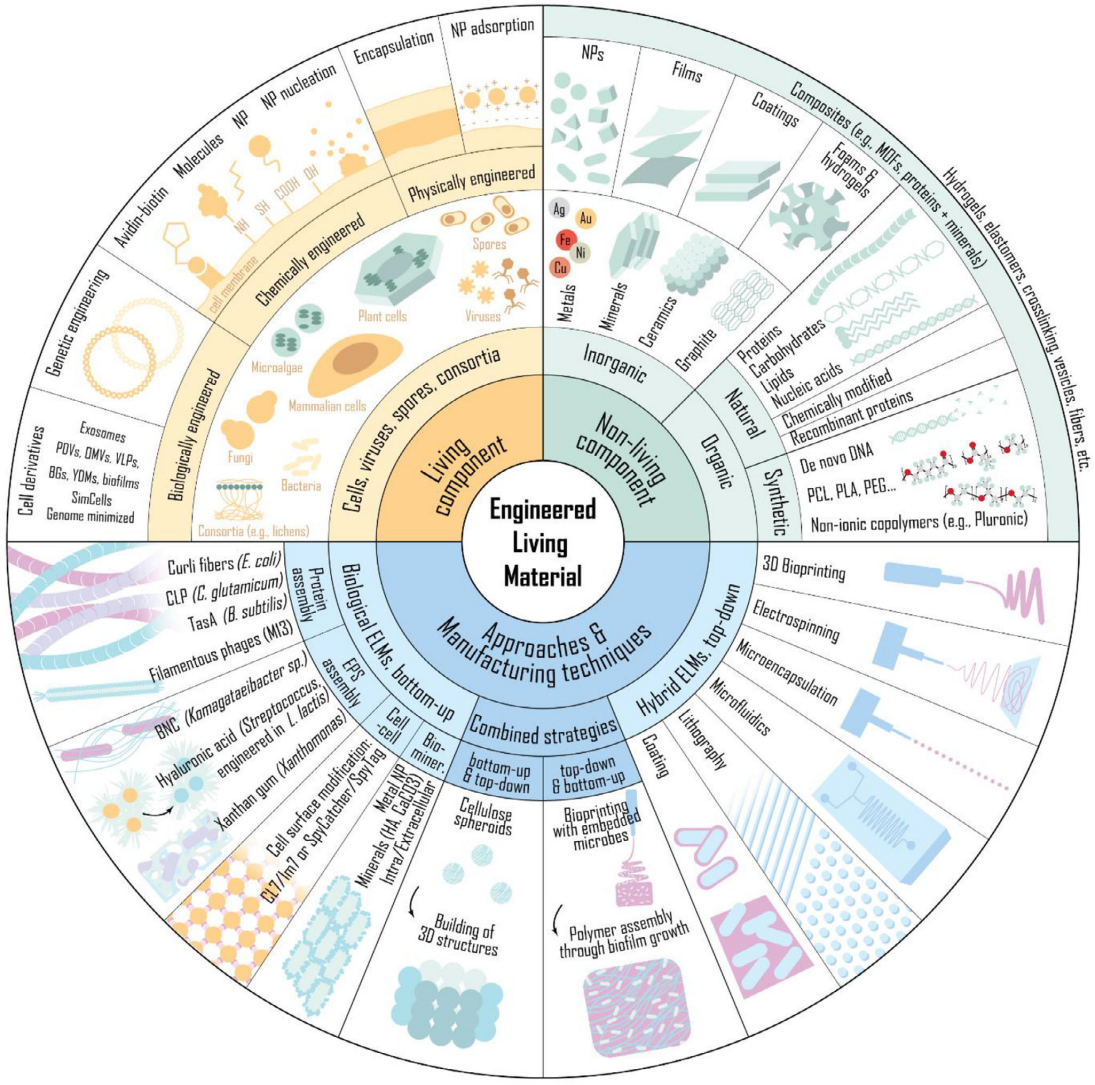
Schematic diagram of ELM components and manufacturing techniques. The living component comprises different cell types, consortia, spores, and viruses, while the non‐living component can be inorganic or organic, natural or synthetic. ELMs can be classified as biological‐ELMs or hybrid‐ELMs according to the approach employed to obtain them—i.e., bottom‐up or top‐down, respectively—and both approaches can be combined to obtain more complex structures. The living component can be engineered prior to performing the desired manufacturing technique or to successfully implement the desired approach. Alternatively, it can be engineered by means of a particular manufacturing technique. PDVs, plant‐derived vesicles; OMVs, outer membrane vesicles; VLPs, virus‐like particles; bacterial ghosts, BGs; YDMs, yeast‐derived microparticles; NP, nanoparticle; MOF, metallic organic framework; PCL, polycaprolactone; PLA, polylactic acid; PEG, polyethylene glycol; CLP, covalently linked pili; HA, hydroxyapatite.

ELMs consist of a living cellular component embedded within a non‐living matrix using either bottom‐up or top‐down approaches (**Figure** **1**). In bottom‐up approaches, the matrix is produced by the living cells themselves, whereas in top‐down approaches, it is added to the living cells separately. The taxonomy of ELMs has been thoroughly discussed in other reviews^[^ ^8^ ^]^ and is under constant evolution as new living materials are developed. Regardless of their classification, the living cellular component functions as the key active element of the ELM and is typically engineered to sense and respond to specific environmental cues. The non‐living component is designed to enhance cell viability, provide structural support, facilitate targeted and on‐demand delivery, compartmentalize cells,^[^ ^9^ ^]^ or provide additional functionalities, such as magnetic properties,^[^ ^10^ ^]^ tunable mechanical properties,^[^ ^11^ ^]^ or material shape changes in response to specific inputs.^[^ ^12^ ^]^ All these features endow ELMs with a wide range of applications in fields like construction, biosensing, energy conversion, bioremediation, biocatalysis, material synthesis, while complying with sustainability.^[^ ^2^, ^13^ ^]^ Given the fast growth of this concept, ELMs are often named accordingly to their field of applicability, leading to Living Building Materials in construction,^[^ ^2^ ^]^ Living Synthelectronics and Hybrid Living Carbon Materials in bioelectronics,^[^ ^13^, ^14^ ^]^ and Living Biointerfaces or Living Therapeutics in biomedicine,^[^ ^15^ ^]^ among others.

#### 1.1.1 The Living Component of ELM

The selection of the living component depends on the final application of the ELM, but is usually based on living cells, mainly fungi, archaebacteria and eubacteria, algae, mammalian and plant cells, or as a consortium.^[^ ^8^, ^16^ ^]^ Mammalian cells have been mostly employed for tissue regeneration,^[^ ^17^ ^]^ to develop drug testing platforms, cultured meat,^[^ ^18^ ^]^ and artificial skin,^[^ ^18b^ ^]^ which requires different cell extraction sources. Nevertheless, advances in microbial synthetic biology have led to the development of novel and improved genetic engineering tools that are increasingly accessible, robust, and easy to implement, making them “easy‐to‐program” responsive cell factories. These properties, in addition to their fast growth rate and ease of handling, make microbes the perfect candidates for ELM development. Despite some recent advancements in the use of consortia,^[^ ^16a^ ^]^ ELMs mostly employ a single strain. The use of engineered consortia within a synergistic approach is still a field that holds great potential to build systems with a higher level of complexity. For example, based on the kombucha consortium, bacteria and genetically modified yeasts can be combined to obtain a bacterial nanocellulose (BNC)‐based ELM with tunable mechanical properties that rely on chemical and optical stimuli.^[^ ^19^ ^]^ Another example that has not been widely explored but holds great potential is the development of synthetic lichens, combining cyanobacteria and fungi (e.g., *Nostoc* and *Aspergilli*) to increase biomass production.^[^ ^20^ ^]^

Looking further into the classification of living systems, viruses and cell derivatives, such as minicells, plant‐derived vesicles (PDVs), cytoplasmic membrane, exosomes, cell‐derived nanovesicles, extracellular vesicles (EVs), bacteria outer membrane vesicles (OMVs), biofilms, virus‐like particles (VLPs), bacterial ghosts (BGs), and yeast‐derived microparticles (YDMs), lack the ability to maintain vital functions. Thus, biomaterials based on these derivatives are categorized as Natural Living Materials or “synthetic cells” rather than living components.^[^ ^6^, ^21^ ^]^ However, they can still perform as smart/responsive elements and be combined with another material, falling under the ELM category. In this regard, the bacteriophage M13 has been genetically modified to develop biosensors;^[^ ^22^ ^]^ chitosan‐coated engineered minicells derived from *Escherichia coli* Nissle 1917 (EcN) can successfully deliver doxorubicin to treat lung carcinoma;^[^ ^23^ ^]^ BGs derived from *E. coli* ATCC 25 922 can be coated with cancer cell membranes and loaded with liposomal paclitaxel to treat metastatic lung cancer;^[^ ^24^ ^]^ and PDV‐like nanoparticles can be used as therapeutic agents and drug carriers due to their low immunogenicity.^[^ ^25^ ^]^ In addition to these cell derivatives, bacterial spores, genome‐minimized strains, and chromosome‐free bacteria cells (SimCells) can also be used to obtain ELMs.^[^ ^26^ ^]^

#### 1.1.2 Non‐Living Components of ELMs

The non‐living component serves as a scaffold whose main role is to provide a stable structure and a suitable environment to support the living component function. Over the last decades, a wide variety of advanced materials have been developed, which, when combined with the living part, enable the incorporation of new or improved features: more sophisticated and targeted delivery systems, longer shelf life, or other desirable biological effects. In this regard, non‐living components are classified into inorganic, organic, and composite materials, with natural or synthetic origin.

*Inorganic Components*. These materials exhibit high mechanical strength, magnetic, electrochemical, and optical properties, and high stability. They consist of metals, minerals, ceramics and glass, and graphite in the form of nanoparticles, films, coatings, foams, and hydrogels. Inorganic components in the form of nanoparticles are often incorporated into the cell surface through chemical reactions or physical interactions for different purposes: iron oxide nanoparticles (NPs) can be attached to a probiotic surface to treat anemia or combined with gold NPs to confer magneto‐optical properties for hyperthermia cancer therapy,^[^ ^27^ ^]^ and rifampicin‐loaded mesoporous silica NPs coated with OMVs from *E. coli* work as a delivery system to treat bacterial infections.^[^ ^28^ ^]^ Although inorganic components are often incorporated as NPs, other structures such as sponges can also be of interest, e.g., graphene oxide sponges loaded with *Shewanella oneidensis*, a bacterium with the ability to efficiently reduce metal ions, can function as bioanodes for sustainable energy generation.^[^ ^29^ ^]^

*Organic Components*. Organic materials offer high biocompatibility and a suitable environment for the living component. Polymers are the biggest subset, including natural proteins (collagen, silk fibroin), polysaccharides (cellulose, alginate, agar, hyaluronic acid, chitin), lipids, and nucleic acids. Natural polymers can be chemically modified to obtain new structures with different features: chitin can be deacetylated to obtain chitosan, cellulose can be modified to yield carboxymethyl cellulose, and gelatin, hyaluronic acid, and alginate can be methacrylated, among others. In turn, new polymers can be obtained using synthetic approaches, such as de‐novo synthesis of DNA sequences (DNA‐based gels, DNA origami), non‐ionic copolymers like Pluronic F‐127,^[^ ^30^ ^]^ poly(ε‐caprolactone) (PCL), polylactic acid (PLA), and polyethylene glycol (PEG) diacrylate. Most of these polymers assemble as networks with structural stability and high water‐holding capacity, giving rise to porous hydrogels.^[^ ^31^ ^]^ These polymer‐based matrices display excellent biocompatibility and tunability in terms of microscale and macroscale organization, supporting cell functions, enhancing cell viability, creating chemical gradients, mechanical confinement, and spatial distribution, while eventually providing new functionalities (such as stretchable or adhesive properties),^[^ ^32^ ^]^ allowing the modulation of cell behavior by means of chemical and physical cues.

Moreover, the living component can be engineered to synthesize polymers or enzymes tailored to generate, reinforce, repair or degrade the matrix. In this regard, recombinant proteins offer several beneficial functions such as cross‐linking, drug loading, responsive behavior, and interactions with other biomolecules and even minerals and ions. Thus, genetic engineering targeting peptides and proteins paved the way for designing matrices that interact specifically with molecules and cells, as discussed in the next section.

The combination of polymers and inorganic compounds gives rise to composites, such as metallic organic frameworks (MOFs) that can be used to wrap and protect anaerobic bacteria,^[^ ^33^ ^]^ and ferromagnets embedded in PVA hydrogels along with engineered bacteria to endow the material with magnetic properties.^[^ ^34^ ^]^ Moreover, glucose polymer‐conjugated silicon NPs loaded with indocyanine green can be internalized by some bacteria through the ABC transporter, resulting in ‘Trojan’ bacteria that can take therapeutics across the blood‐brain barrier to treat glioblastoma.^[^ ^35^ ^]^

#### 1.1.3 ELM Manufacturing

ELMs can be designed using two main strategies. Bottom‐up methods take advantage of natural polymers synthesized by living organisms, which are responsible for performing essential functions such as providing structural support to cells, protection against environmental threats, and cell energy storage. On the other hand, top‐down approaches involve the encapsulation of living organisms within exogenous materials.^[^ ^4b^ ^]^

*Bottom‐up Approach*. This strategy takes inspiration from natural biological systems, such as wood, bone and microbial biofilms, in which cells can autonomously build a self‐organized material that senses and responds to environmental cues, can be remodeled, and is able to grow, self‐heal, and evolve. Hence, bottom‐up approaches often employ bacteria and fungi due to their ease of culture, fast growth rate, and ability to produce extracellular polymeric matrices relatively readily compared to mammalian and plant cells.^[^ ^8b^ ^]^ The four main methods are the protein‐based assembly, exopolysaccharide‐based assembly, cell‐to‐cell attachment,^[^ ^36^ ^]^ and biomineralization.^[^ ^37^ ^]^ One of the best‐known examples of protein‐based assembly are Curli fibres. Some bacterial species from *Enterobacteriaciae* and, most notably, *E. coli*, produce biofilms based on amyloid curli nanofibers, also known as curli pili, whose main protein building block is CsgA. CsgA is secreted into the media, and the cell‐surface CsgB protein triggers its nucleation and further polymerization, leading to self‐assembled fibrillar networks resistant to several harsh conditions (heat, pH, solvents, etc.). CsgA monomers can be engineered to obtain healable bioplastics,^[^ ^38^ ^]^ combined with other polymers to develop living bioadhesives,^[^ ^39^ ^]^ or chemically modified to obtain conductive self‐healing films.^[^ ^40^ ^]^ Indeed, curli expression can also be implemented in other bacterial chassis like *Pichia pastoris*, and CsgA can be fused to proteins in their C‐terminus to obtain chimeric bio‐adhesive proteins.^[^ ^41^ ^]^ Other proteins such as covalently linked pili monomers from *Corynebacterium glutamicum*, have been fused with different cellulose‐degrading enzymes and, similarly to curli, can self‐assemble in the extracellular space, generating a biofilm that can convert cellulosic waste into valuable compounds.^[^ ^42^ ^]^ On the other hand, the non‐amyloid TasA protein from *Bacillus subtilis* ^[^ ^43^ ^]^ has been fused to Mefp3 and Mefp4 (adhesive mussel foot proteins derived from *Mytilus edulis*), PETase (poly(ethylene terephthalate) (PET) hydrolase), and the organophosphate hydrolase, among others.^[^ ^44^ ^]^ The filamentous bacteriophage M13—which is a semiflexible protein nanofilament—can also be chemically modified and genetically engineered to obtain bioactive hydrogels and liquid crystals.^[^ ^45^ ^]^

Regarding carbohydrate self‐assembly, one of the most outstanding examples is the use of BNC. This polymer is produced by different bacteria as part of their biofilm, most notably by *Komagataeibacter* sp., and recent studies are setting the basis for developing genetic tools to precisely regulate cellulose fermentation.^[^ ^46^ ^]^ Compared to protein expression, carbohydrate fabrication is significantly more complex: the synthetic pathway involves different enzymes, regulatory elements, and a secretion mechanism. Hence, engineering a cell to produce a modified exopolysaccharide is substantially more challenging, yet some studies reported the successful engineering of *Xanthomonas campestris* to modify the mechanical properties of xanthan gum,^[^ ^47^ ^]^ and the cloning of the whole hyaluronic acid synthetic pathway from pathogenic species into safer bacterial hosts, such as *Lactococcus lactis*.^[^ ^48^ ^]^

Direct cell‐to‐cell binding using the “living component” by itself allows the creation of stable structures, e.g. by genetically engineering four populations of *Saccharomyces cerevisiae* to express SpyCatcher, SpyTag, CL7, and Im7 on their surface.^[^ ^49^ ^]^ When pairs of yeast populations expressing SpyCatcher/SpyTag or CL7/Im7 are mixed, the cells spontaneously assemble into a high‐order structure. In addition to cell‐to‐cell binding, structures from the nano‐ to macroscale can also be obtained through biomineralization processes.^[^ ^50^ ^]^ Several organisms can naturally drive the spontaneous biomineralization of ions into NPs and higher‐order structures, such as bone. This nucleation process can be catalyzed by an enzyme or can start in a nucleation site located intracellularly (magnetite in magnetotactic bacteria)^[^ ^51^ ^]^ or extracellularly (often in an extracellular matrix or cell membrane protein). Microbial natural carbonate biomineralization ability can be used to reinforce materials,^[^ ^52^ ^]^ and also the use of collagen matrices allows hydroxyapatite (HA) nucleation while embedding probiotics to obtain functional foods.^[^ ^53^ ^]^ Proteins comprising nucleation sites can also be implemented in other organisms, e.g., HA‐binding peptide fused to *E. coli* Curli monomers,^[^ ^54^ ^]^ and Mms6, a peptide for magnetite templating, fused to *B. subtilis* TasA protein.^[^ ^44^ ^]^ Interestingly, this approach allows the formation of minerals with specific size, shape, and phase, which endows ELMs with new chemical, physical, optical or mechanical properties. All the above living‐assembly approaches are often combined with manufacturing techniques like moulding, casting, and 3D printing.

*Top‐Down Approach*. Conversely, top‐down approaches combine the living component with previously extracted or chemically synthesized materials. 3D bioprinting, coating, microencapsulation, microfluidics, lithography, and electrospinning are some of the most employed manufacturing techniques. These techniques can be customized to allow the integration of cells in different configurations: embedded within the material,^[^ ^16b^ ^]^ in a layer‐by‐layer structure,^[^ ^55^ ^]^ contained in a hollow space or chamber‐like configuration,^[^ ^32^, ^56^ ^]^ or adsorbed onto the surface of the bulk material.^[^ ^15a^ ^]^ These manufacturing techniques have been extensively discussed in other works and are outside the scope of this review.^[^ ^1^, ^57^ ^]^

Many pioneering efforts have focused on the modification and optimization of a single manufacturing technique to obtain advanced ELMs, although there are some emerging strategies that combine both top‐down and bottom‐up approaches.^[^ ^58^ ^]^ In 2021, the obtention of BNC spheroids (bottom‐up) to further build 2D and 3D structures (top‐down) was described,^[^ ^59^ ^]^ but also a bioink comprising *Komagataeibacter xylinus* that can be 3D printed (top‐down) and further incubated to grow a cellulose‐based scaffold (bottom‐up).^[^ ^60^ ^]^ In another study, the fungus *Ganoderma* sp. was embedded in lignocellulosic material (top‐down), allowing the growth of mycelium and the binding together of the feedstock (bottom‐up) to obtain human‐scale structures for construction.^[^ ^61^ ^]^

Therefore, depending on the manufacturing method, the living component can be subjected mainly to chemical or physical engineering, while biological engineering is usually performed prior to the implementation of a manufacturing technique (**Figure** **1**). In this regard, most bottom‐up approaches take advantage of the polymer synthesis or biomineralization ability naturally present in some organisms and viruses to obtain matrices, which results in physical (encapsulation) and chemical (nanoparticle nucleation) engineering, respectively. Additionally, the living component can be chemically or biologically engineered to accommodate bottom‐up methodologies, allowing for cell‐to‐cell attachment or for producing self‐assembling proteins/exopolysaccharides. In contrast, top‐down approaches generally focus on encapsulation of the living component in inorganic or organic polymeric matrices, falling under the category of physically engineered. Before undergoing top‐down manufacturing, the organism can be biologically, chemically or physically engineered (e.g., genetically engineered to sense and produce an active compound by attaching a desired molecule on their surface that recognizes extracellular cues, or after adsorbing nanoparticles on their surface). Hence, to obtain the desired performance of the ELM, the selection of the manufacturing technique should align with a suitable cell engineering approach, before or after its implementation.

### 1.2 Engineering the Living Features of ELMs

All these naturally occurring properties in living systems, combined with the wide range of potential modifications to obtain advanced or new functionalities, make ELMs particularly promising for targeting health conditions. In this regard, synthetic biology has emerged as a valuable tool to program the cellular behavior by designing genetic circuits, based on functional units with defined inputs and outputs.^[^ ^13^, ^15^ ^]^ Genetic circuits can be divided into three basic modules: the input module detects a signal (biotic or abiotic, intracellular or extracellular) and transforms it into a molecular response; the operation module detects the response from the input module and determines the cellular behavior; and finally, the output module generates the desired response. For instance, the input could be the binding of a ligand to a membrane receptor that triggers a phosphorylation, which activates a transcription unit that ultimately targets a gene and synthesizes a protein as an output. The genetic circuit may comprise the three basic modules or may expand its level of complexity by adding further modules (e.g., sensing different cues, following Boolean logic gates, triggering the activation of self‐inducible systems, involving the synthesis of several proteins or enzymes that trigger a multi‐step cascade, etc.).^[^ ^62^ ^]^ Such inducible systems are based on the use of efficient inducers that finely control the response of microorganisms in a dose‐dependent and spatiotemporal manner. These cues specifically regulate a promoter's transcriptional activity, being small‐molecule inducible systems one of the most exploited, yet other systems can sense oxygen, changes in the pH^[^ ^63^ ^]^ or in the temperature,^[^ ^64^ ^]^ or can be activated by light irradiation.^[^ ^65^ ^]^ Sensing systems are naturally present in living systems and can be translated from one organism to another or be synthetically designed.^[^ ^66^ ^]^

Focusing on therapy, both traditional and more sophisticated systems can be adapted to treat different targets and conditions: in topical applications, the ELM can be induced by the exogenous addition of small molecules, while for other less accessible tissues, ELM activation can rely on changes in the biochemistry of their surroundings or the presence of disease biomarkers. However, using microbes to treat a target tissue or organ that is not the niche of such organisms may also decrease the effectiveness of the treatment, adversely impact the local microbiome,^[^ ^67^ ^]^ trigger non‐desirable biological responses, or even result in pathogenic behaviors.^[^ ^68^ ^]^ As an alternative, probiotics arise as a safer platform for designing engineered materials as living therapeutics. The following sections discuss the use of probiotics to develop PLMs for therapeutic applications, focusing on genetic engineering tools, recent advances, biosafety challenges, and regulatory frameworks.

## 2 Probiotic Living Materials in Therapy

### 2.1 Biomedical Applications of Probiotic‐Based Materials

This section focuses on the advancements in the use of native probiotics (i.e., non‐genetically modified) as therapeutics. The World Health Organization (WHO) and International Scientific Association for Probiotics and Prebiotics (ISAPP) defines probiotics as “live microorganisms that, when administered in adequate quantities, confer a health benefit on the host”.^[^ ^69^ ^]^ Specifically, the health benefits conferred by a probiotic must have been validated in properly controlled studies, excluding live cultures associated with fermented foods and undefined fecal microbiota transplants. Likewise, commensal organisms cannot be considered as probiotics unless they have been isolated, characterized, and their health benefits substantiated. Furthermore, the United States Food and Drug Administration (FDA) instructs that any food or food additive must be proven to be sufficiently safe under the conditions of its intended use by recognized experts before entering the market. This status is known as Generally Recognized As Safe (GRAS).^[^ ^70^ ^]^ Consequently, commonly used probiotic microorganisms have GRAS status. These microorganisms are usually cultivars derived from human microbiota and belong to the genera *Lactobacillus*, *Bacillus*, *Bifidobacterium*, and *Saccharomyces*.

**Figure 2:**
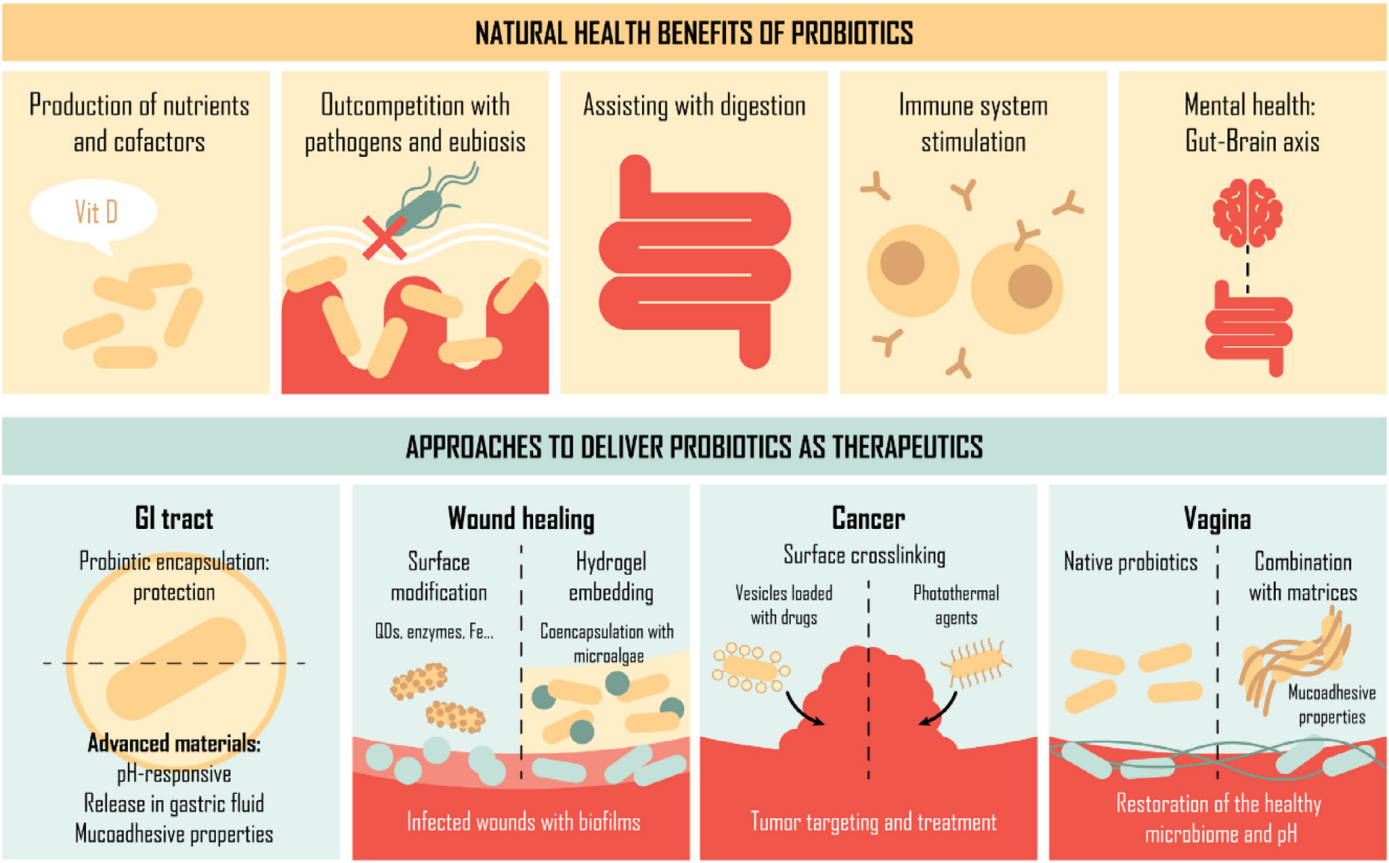
Schematic diagram of the health benefits of probiotic microorganisms, and common approaches to facilitate their delivery. Probiotics exert many health benefits of probiotics through their presence, including the production of vitamins and minerals, their natural antimicrobial properties, helping digestion, and their influence on the immune system and the gut–brain axis. Probiotics can be used as therapeutics for specific diseases or wounds, and strategies, such as encapsulation, surface modification, or incorporating them in matrices, enhance their efficacy by protecting them from the harsh environment of the body.

Probiotics primarily promote health by supporting a balanced gut microbiota to benefit digestion and immunity (**Figure** **2**). Probiotics exert their beneficial properties through multiple mechanisms, including the production of vital nutrients and cofactors (such as vitamin D),^[^ ^71^ ^]^ assisting with mineral absorption,^[^ ^72^ ^]^ normalization of disturbed microbiota, out‐competition of pathogens, assisting with digestion,^[^ ^73^ ^]^ and immune system stimulation.^[^ ^74^ ^]^ However, improved gastrointestinal (GI) health is not the only beneficial effect probiotics can exert. The therapeutic effect of probiotics is being investigated as treatments of cancers,^[^ ^75^ ^]^ to alleviate osteoporosis,^[^ ^76^ ^]^ assist wound healing,^[^ ^77^ ^]^ combat vaginal infections,^[^ ^78^ ^]^ and even the so‐called psychobiotics, a class of probiotics that exert anxiolytic and antidepressant effects by addressing imbalances in the gut microbiota to influence the gut‐brain‐axis and improve mental health.^[^ ^79^ ^]^ Although some high‐quality trials support the benefits of probiotics, contradictory findings lead to conflicting and inconclusive overall results.^[^ ^74^ ^]^ One reason for this is that probiotics often need to reach difficult‐to‐access areas, requiring them to travel through the body and endure harsh environments like the GI tract. Many probiotics are damaged by the stomach's low pH or bile salts in the intestine, and anaerobic strains such as Bifidobacterium are sensitive to oxygen and lack the enzymes to protect themselves from reactive oxygen species (ROS).^[^ ^80^ ^]^ Consequently, it is difficult to assess whether a sufficient number of viable cells endured to provide a therapeutic benefit. Therefore, protecting probiotics by combining them with materials can enhance their survival, ensure adequate delivery of live microbes, and enable controlled release.

*GI Tract*. To protect probiotics from harsh conditions and enhance their viability, researchers have integrated them with various materials. Microencapsulation can improve probiotic survival and enable targeted release within the GI tract. This technique involves entrapping probiotics within polymer capsules, creating a protective microenvironment and functional barrier against the external environment. Microgels composed of natural biopolymers such as alginate and chitosan have been used for probiotic encapsulation,^[^ ^81^ ^]^ and synthetic polymers such as polyvinyl alcohol can be used to produce food‐grade chewable gummy sweets as probiotic carriers.^[^ ^82^ ^]^ Nanostructured biopolymers, such as cellulose nanocrystals and cellulose nanofibers, have also been employed to encapsulate probiotics for GI tract delivery due to their high crystallinity and biocompatibility.^[^ ^83^ ^]^ Over time, material platforms have evolved beyond simple protection to possess additional functions. For instance, pH‐responsive composite alginate/cellulose macrospheres could encapsulate *Lactiplantibacillus plantarum* and swell in response to simulated gastric fluid to release the probiotic, facilitating intestine‐targeted delivery,^[^ ^84^ ^]^ and chitosan‐based systems can disassemble to release the probiotic.^[^ ^81^, ^85^ ^]^ Mucoadhesive properties can be incorporated, for example, by including cationically‐charged chitosan allows for electrostatic interactions with mucus.^[^ ^86^ ^]^ Such responsive systems enhance therapeutic efficacy by reducing the frequency of treatments and minimizing off‐target effects.

*Wound Healing*. The skin is a physical barrier that acts as the body's first line of defense against infectious pathogens and is colonized by a diverse microbiota involving bacteria, fungi, archaea, mites, and viruses across multiple microenvironments.^[^ ^87^ ^]^ Microbes of these communities can enter the body through open wounds and influence the wound healing process. Open wounds are vulnerable to infection by pathogens like Staphylococcus aureus, Pseudomonas aeruginosa, Candida albicans, and E. coli, which can form tough biofilms that hinder the healing process and make treating with antibiotics difficult. Some commensal microbes can interact with the immune system and promote healing by reducing inflammation^[^ ^88^ ^]^ or stimulate immune responses against pathogens.^[^ ^89^ ^]^ As such, integrating probiotics like L. plantarum and Lacticaseibacillus rhamnosus into materials has shown promise as adjuvants to wound healing by suppressing pathogens and modulating inflammation. For example, a hybrid of *L. rhamnosus* modified with MXene quantum dots, lactate oxidase, and Fe^2+^ could disrupt *S. aureus* biofilms and kill the pathogen as a result of a chain reaction: the oxidation of the lactic acid produced by the probiotic released hydroxide radicals, giving rise to thermal energy and antimicrobial ROS after NIR irradiation of the quantum dots.^[^ ^90^ ^]^

However, direct application of probiotics to open wounds can be inefficient and risks exposure to the immune system. Recent efforts have focused on embedding the probiotics in hydrogels to protect them from mechanical and environmental stresses, increase their viability, and control therapeutic release whilst providing a protective barrier around the wound.^[^ ^91^ ^]^ There is keen interest in injectable or bio‐printable living materials for healing acute and chronic (diabetic) wounds, which are slow to heal due to immune stress and are prone to infection due to their hyperglycaemic environment.^[^ ^92^ ^]^ For example, ProGel, an injectable two‐component hydrogel cross‐linked by dynamic Schiff‐bonds that conferred self‐healing to protect the material from mechanical damage, was developed.^[^ ^93^ ^]^ ProGel concentrated *L. plantarum* at the wound site, and demonstrated the release of factors with potent antimicrobial activity against *S. aureus*, and *P. aeruginosa*, and even modest anti‐fungal activity against *C. albicans*, preventing biofilm formation in ex vivo human skin model. Continuous lactic acid released from *L. plantarum*‐loaded hydrogels promoted healthy skin microbiota and suppressed pathogens while alleviating inflammation.^[^ ^94^ ^]^ Others have developed living materials to address hypoxia from damaged vasculature in diabetic wounds. A hydrogel exhibiting antimicrobial probiotic B. subtilis co‐encapsulated with an oxygen‐producing microalgae, Chromochloris zofingiensis, enhanced human dermal fibroblast migration and angiogenesis in vitro.^[^ ^95^ ^]^ In diabetic rats, this living hydrogel diversified the wound microbiome and accelerated healing by upregulating genes for skin regeneration.

*Cancer*. Anaerobic bacteria are known to target and colonize tumors due to the hypoxic tumor microenvironment, where they modulate the antitumor immune response and influence the efficacy of cancer therapies.^[^ ^96^ ^]^ Probiotic‐based materials have been explored as potential adjuvants to typical cancer therapies^[^ ^97^ ^]^ but find it difficult to penetrate the dense tumoral extracellular matrix (ECM). Strategies have been developed to enhance the delivery of probiotics to tumors by remodeling the ECM while carrying therapeutic drugs, enhancing its antitumor efficacy. A probiotic nano‐system was designed to remodel the ECM of pancreatic tumors and penetrate to deliver chemotherapeutic drugs loaded into vesicles cross‐linked onto *Clostridium butyricum*.^[^ ^98^ ^]^ The nano‐system could deliver vactosertib to silence ECM‐producing stellate cells and deliver chemotherapeutic gemcitabine deep into the tumor. Moreover, the probiotic itself exhibited antimicrobial effects against tumor‐colonizing gammaproteobacteria, known to metabolize gemcitabine.^[^ ^96^ ^]^ Likewise, EcN cross‐linked to cypate, a photothermal agent, was shown to enhance phototherapeutic treatments for lung cancer in combination with hyperbaric oxygen treatment.^[^ ^99^ ^]^

*Vaginal Infections*. The main residents of a healthy vaginal microbiome are *Lactobacilli* spp. which create an acidic microenvironment that prevents colonization of the vagina by pathogens.^[^ ^100^ ^]^ Bacterial vaginosis is diagnosed when the microbiota is dominated by other obligate and facultative anaerobes, which increases the chance of infections and affects reproductive outcomes. Consequently, *Lactobacilli* spp. are thought to play a role in regulating vaginal health and alleviating gynecological diseases,^[^ ^78b^ ^]^ and administration of this probiotic has been investigated to treat bacterial vaginosis, fungal vulvovaginal candidiasis, aerobic vaginitis, and other viral infections.^[^ ^78a^ ^]^ Yeast‐based probiotics, such as live *S. cerevisiae*, can also fight candidiasis by reducing the adherence of infectious *Candida* to the mucosal surface, suppress key virulence factors, and stimulate the host immune system to produce antimicrobial ROS.^[^ ^101^ ^]^ However, despite promising in vivo results, clinical trials involving the use of probiotics to treat vaginal infections have been mixed. In part, this is because oral administration exposes the microbe to the digestive tract and takes a long time to reach the vaginal tract. Intravaginal application is effective in less time, but the altered microenvironment present during infection reduces the viability and therapeutic potential of *Lactobacillus*. Living material strategies have been explored to protect and localize probiotics under these harsh conditions. For example, the development of living probiotic material composed of two *Lactobacillus* strains entrapped with a collagen scaffold supported the growth of probiotic biofilms and enhanced their adhesion to simulated vaginal mucosa, whilst restoring it to the healthy vaginal pH.^[^ ^102^ ^]^

There is great potential and keen interest in exploiting the pro‐health benefits of probiotics to treat or alleviate symptoms of disease across many microenvironments in the body. Although there have been efforts to toughen or protect probiotics within material platforms to improve their viability and efficacy, other strategies to enhance their therapeutic properties focus on the use of synthetic biology methods to genetically engineer probiotics with tailored functions and behaviors.

### 2.2 Genetic Engineering Tools for Key Probiotics

Synthetic biology has emerged as a valuable tool to engineer probiotics with advanced or new functionalities that, in combination with their inherently health‐promoting properties, allow the design of promising PLMs for targeting health conditions. The Registry of Standardized Parts, a catalog of characterized DNA sequences, helps with the bioengineering of microorganisms but is biased toward *E. coli*. Standardization speeds up the design‐build‐test cycle and enables the utilization of common parts by different users. Most complex bioengineering strategies employ Golden Gate assembly,^[^ ^103^ ^]^ using type II restriction enzymes, which cut outside of their recognition sites, so that the resulting overhangs between these positions can be any designed sequence. BioBricks employs a defined set of DNA overhangs to be released post‐digestion to direct the order of parts when assembling transcriptional units.^[^ ^104^ ^]^ Such a system permits one‐pot, unidirectional assembly of genes or multi‐gene constructs, with the programmable DNA overhangs dictating the order of assembly. Since the advent of Golden Gate assembly,^[^ ^103^, ^105^ ^]^ many hierarchically structured, standardized genetic toolkits have been developed for several model probiotic organisms. The Modular Cloning system (MoClo) for the production of eukaryotic multigene constructs was the first of these toolkits developed.^[^ ^106^ ^]^ The structure of these toolkits provides a pipeline for the construction of complex, multigene constructs from a library of validated genetic components and three levels of vectors. Level 0 vectors host the individual genetic components, such as promoters, ribosome binding sites (RBSs), N‐ or C‐terminal protein tags, and terminators. At level 1, the individual genetic components are assembled into transcriptional units through the Golden Gate method. Subsequently, individual transcriptional units can be combined into multigene constructs at level 2 using the same strategy. Usually, the assembly and amplification processes are performed in lab *E. coli* strains, so the final step is usually to export the final gene construct into a shuttle vector compatible with the host organism. Additionally, many toolkits also include options to integrate the assembled constructs into the genome of the host organism. The following section will highlight genetic engineering tools and toolkits that have been applied to probiotic microorganisms (**Table** **1**).

**Table 1:** Genetic engineering tools and toolkits for probiotic organisms.

Microorganism	Genetic tool or kit	Function or purpose	Refs.
*Saccharomyces cerevisiae*	Yeast toolkit (YTK)	Hierarchically structured toolkit of characterized parts to streamline genetic engineering	[109]
	Split synthetic zinc‐finger transcription factor	Protein expression in response to light (470 nm)	[110]
	G‐protein coupled receptor (GPCR)‐based cell sensing	Five characterized GPCRs to engineer cells to induce protein expression in response to extracellular peptides, hormones, or metabolites	[111]
	yeast Tunable Expression System Toolkit (yTEST)	Five standardized inducible promoters regulated by small molecules	[112]
	Framework for producing guide RNA arrays	Expanded YTK with to simplify the production of guide RNAs for CRISPR/Cas9 applications	[113]
	Yeast Multiplex Toolkit (MYT)	Hierarchically structured toolkit of characterized parts to streamline genomic integration of synthetic gene circuits	[114]
*Saccharomyces boulardii*	Toolkit of characterized genetic components	Hierarchically structured toolkit of characterized parts to streamline genomic integration of synthetic gene circuits	[117]
*Bacillus subtilis*	SubtiToolKit (STK)	Hierarchically structured toolkit of characterized parts to streamline genetic engineering	[118]
*Lactiplantibacillus plantarum*	Optimized in vitro cloning	A direct cloning method that avoided the need for an intermediate host to assemble DNA	[122]
	Phage‐derived promoter pTec	Stronger promoter for protein expression	[123]
	Promoter, pTlpA, from distantly related species	Strong promoter for protein expression	[123]
	Toxin/anti‐toxin selection system	Avoids the need for antibiotic selection	[123b]
	Signal peptides for protein secretion	Optimized to secrete high levels of expressed proteins	[124]
	pSIP vector suite	Modular vector for tightly controlled recombinant protein expression	[125]
*Lactococcus lactis*	Nisin‐inducible promoter (NICE)	Inducible protein expression in response to the polycyclic peptide nisin	[131]
	pNZDual plasmid	Two recombinant proteins can be expressed upon nisin induction	[133]
	pDawn promoter	Light‐inducible protein expression (475 nm), employed in *L. lactis*	[85]
	*L. lactis* NZ9000‐4	Strain exhibiting higher protein expression levels owing to genome reduction	[136]
	Thymidine synthetase‐deficient strain	Auxotrophic selection, no antibiotic selection needed	[137]
	Highly active recombinase, RecT	Efficient genomic integration of synthetic gene constructs, and can make points mutations and deletions	[138]
	Secretion peptide sequence from Usp45	Genetically fused signal peptide to direct the secretion of expressed proteins	[140]
	pSEUDO plasmid	Genomic integration of synthetic genes	[154]
*Escherichia coli* Nissle 1917	Genomic integration of T7 RNA polymerase	Allowed recombinant protein expression from common vectors, such as pET	[146]
	Thermally activated protein expression, pTlpA	Protein expression is induced at 37 °C and above, but repressed below	[149]
	NorR, protein expression in response to nitric oxide	Inducible expression upon the detection of a disease biomarker	[151]
	Dual auxotrophic‐ and essential gene selection	Pipeline for assembling plasmids without the need for antibiotic‐resistance genes	[152]
	Split ccdA/ccdB toxin/antitoxin system	Avoid the need for antibiotics. The poison is integrated in the genome, and the antitoxin is located on the vector	[153]
	PRO_3_TECT	Protein secretion toolkit	[157]
	pMUT	Domesticated cryptic plasmids found endogenously in clinical samples	[156]

*Saccharomyces*. Owing to the efficient and well understood homologous recombination system in *S. cerevisiae*, many toolkits have been developed to engineer *Saccharomyces*, as reviewed elsewhere.^[^ ^107^ ^]^ Earlier toolkits were limited in their applicability^[^ ^108^ ^]^ until MoClo was adapted to make the Yeast Toolkit (YTK).^[^ ^109^ ^]^ YTK is a flexible platform comprised of a diverse library of 96 genetic components that were validated or optimized in *S. cerevisiae* to facilitate quick and easy plasmid construction and provided two strategies to enhance the efficiency of genome integrations. The toolkit is also available from Addgene (Kit #1 000 000 061). As a result, YTK has been adopted in many *S. cerevisiae* engineering investigations, including developing optogenetic circuits^[^ ^110^ ^]^ and GPCR‐based cell sensing,^[^ ^111^ ^]^ and expanded on with a set of standardized inducible promoters^[^ ^112^ ^]^ and a framework for constructing gRNA arrays and enhancing the efficiency of combinatorial assemblies.^[^ ^113^ ^]^ Recently, the capabilities and flexibility of YTK were expanded upon in the Multiplex Yeast Toolkit (MYT).^[^ ^114^ ^]^ Ten new integration loci, that were sufficiently distanced from endogenous open reading frames, were characterized and ten new vectors for markerless (lacking a selection marker) genome integration were provided, along with an efficient CRISPR‐Cas9 toolkit to support the simultaneous integration of all ten integration vectors with high efficiency in a single step. The toolkit can construct more complex synthetic gene circuits than YTK, involving up to ten transcription units instead of six, in less time. A simplification of the level 1 cassettes allows users to design large multigene constructs and assemble them directly into the yeast genome bypassing level 2 plasmids. To provide additional flexibility to MYT, the included integration sites are conserved across multiple strains of *Saccharomyces*. MYT is also available to purchase from Addgene (Kit #1 000 000 229).

Owing to their genomic similarity, bioengineering strategies previously developed for *S. cerevisiae* have been successfully applied to the probiotic *S. boulardii*, including vectors^[^ ^115^ ^]^ and small‐molecule inducible promoters.^[^ ^116^ ^]^ YTK has also served as a blueprint for the development of a synthetic toolkit for *S. boulardii*, where genetic elements common to both *S. cerevisiae* and *S. boulardii* were characterized in *S. boulardii*, revealing that many elements from *S. cerevisiae* behave predictably in *S. boulardii*. For example, 16 out of 18 constitutive promoters, exhibiting over 97% sequence identity between the two species, provided similar protein expression levels, and the other two gave 10‐fold higher expression, providing a means to tune target protein expression.^[^ ^117^ ^]^ Moreover, an efficient genome integration strategy was provided with the assistance of CRISPR‐Cas12a. Although this toolkit is still limited in its complexity, these findings provide hope that the *S. boulardii* genetic editing toolkit can be expanded or integrated with the other yeast toolkits to expedite the bioengineering of *S. boulardii* with complex genetic circuits.

*Bacillus*. Despite the availability of genetic toolkits for multiple model organisms, Gram‐positives such as *B. subtilis* exhibited only a few modular tools for DNA assembly and genome integration, and they were not either standardized or efficient to be considered for widespread use among Gram‐positives. Recently, the SubtiToolKit (STK) was developed to facilitate rapid bioengineering of *B. subtilis*.^[^ ^118^ ^]^ Similar to the yeast toolkits described above, STK is a highly modular and standardized toolkit with a simple hierarchical structure that can be used to engineer *B. subtilis* and other Gram‐positive bacteria. After assembling transcription units and multigene constructs in *E. coli* in the level 0, 1, and 2 plasmids, they are then transferred to a series of domesticated shuttle vectors able to replicate in *B. subtilis*. The high efficiency of STK facilitated combinatorial assembly reactions, a one‐pot assembly involving a mixture of all library components and a single backbone to produce all possible combinations of parts. Such combinatorial assemblies are useful to provide a large range of expression efficiencies from a relatively small number of building blocks to reduce the number of characterized components required in the final library. This effort is simple in *E. coli*, but much more difficult in *B. subtilis*, highlighting a bottleneck in assessing the quality of efficient genetic circuit construction. STK can also facilitate genomic integration of assembled linear gene circuits and offers a novel *E. coli* expression blocking device to assemble and screen protein constructs harbouring *B. subtilis* secretion tags, which are toxic to *E. coli* and make Golden Gate assembly problematic. This blocking device is a double terminator flanked by two *B. subtilis* recombination sites inserted before the RBS to prevent transcription in *E. coli*, but when transferred to *B. subtilis*, native recombinases remove the terminators allowing full protein expression. Although only recently developed, it is hoped that STK could provide a platform for the development of advanced genetic engineering toolkits for other Gram‐positive bacteria, including probiotic lactic acid bacteria.^[^ ^118^ ^]^

*Lactic Acid Bacteria*. Progress in developing genetic manipulation technologies to engineer Gram‐positive lactic acid bacteria (LAB) to express and deliver heterologous proteins is behind that of other probiotic species. The thick outer cell wall of Gram‐positive bacteria makes it difficult to transform them with exogenous DNA. Bacterial conjugation, the horizontal gene transfer of genetic material from donor to recipient, can be achieved using an *E. coli* donor, and was only recently achieved in many LAB, including *L. plantarum*.^[^ ^119^ ^]^ Alternatively, electroporation is the most efficient way of importing DNA into LAB, but the procedure must be optimized to each strain.^[^ ^120^ ^]^ The most prevalent strategy to bioengineering LABs involves assembling gene constructs in *E. coli* before transferring to shuttle vectors, and purifying these in high quantities to use for transformations, although consideration must be given to genetic differences and restriction enzyme profiles between *E. coli* and LAB. Recently, the genetic editing toolbox available for *L. plantarum* was expanded.^[^ ^121^ ^]^ An optimized and direct in vitro cloning method for amplifying circularized genetic constructs to directly transform *L. plantarum* was devised, which circumvented the need for an intermediate host.^[^ ^122^ ^]^ Furthermore, two new strong promoters that induced higher levels of protein expression than previous promoters,^[^ ^123^ ^]^ toxin/antitoxin systems,^[^ ^123b^ ^]^ and optimized signal peptides for the secretion of high levels of heterologously expressed proteins were characterized.^[^ ^124^ ^]^ Additionally, the pSIP vector suite was developed for tightly controlled inducible recombinant protein expression in *L. plantarum* in response to a peptide pheromone evolved for quorum‐sensing,^[^ ^125^ ^]^ and has been employed to express antigens for tuberculosis, displayed on the bacteria surface.^[^ ^126^ ^]^

*L. lactis* has been the most widely developed LAB for bioengineering, but most attention has focused on improving it as a microbial factory in industrial settings. Simple modifications to its metabolism meant it could produce a range of carbon‐based products,^[^ ^127^ ^]^ although it can be used to produce vitamins and heterologous proteins. Despite its genetic editing toolbox is limited,^[^ ^128^ ^]^ it has been expanded with a host of compatible vectors^[^ ^129^ ^]^ and a range of constitutive and inducible promoters that function in *L. lactis* and are compatible with other LAB.^[^ ^130^ ^]^ The nisin‐inducible system (NICE)^[^ ^131^ ^]^ is the best characterized and most widely used promoter for protein expression in *L. lactis*. It is a quorum sensor with a two‐component system that responds to nisin. Typically, the gene of interest is placed under the control of the PnisA promoter on plasmids, and the associated regulators are genomically incorporated. The complexity of *L. lactis* engineering was improved by re‐engineering variants with different expression strengths^[^ ^132^ ^]^ and designing the plasmid pNZDual,^[^ ^133^ ^]^ where NICE regulates two heterologous proteins, such as ligands for tumor antigens alongside pro‐inflammatory cytokines.^[^ ^134^ ^]^ In addition to inducible promoters, the optogenetic promoter pDawn^[^ ^135^ ^]^ can be used in *L. lactis* to trigger protein expression in response to blue light,^[^ ^85^ ^]^ but requires co‐delivery with up‐conversion materials (UCMs) to function efficiently in vivo. The development of advanced, environmental responsive promoters would enhance the potential of using *L. lactis* in living biotherapeutic materials.^[^ ^127^ ^]^ Regarding bioengineering *L. lactis* as platforms strains, the NZ9000‐4 had four large nonessential regions of the genome removed and exhibited higher heterologous protein expression levels than the wild‐type,^[^ ^136^ ^]^ and a thymidine synthase deficient strain was created to avoid the need for antibiotic selection.^[^ ^137^ ^]^ A rapid and versatile tool for genomic engineering was developed by employing a highly active recombinase to mediate homologous recombination between ssDNA and the *L. lactis* chromosome, and could be used to make point mutations and perform genomic deletions and insertions.^[^ ^138^ ^]^ This tool was used to insert a gene cluster for the synthesis of the antioxidant lycopene into *L. lactis* so it could synthesize the molecule in situ to offer cytoprotective effects to intestinal epithelia cells in vitro.^[^ ^139^ ^]^ Furthermore, heterologously expressed proteins can be secreted from *L. lactis* by genetically fusing a signal peptide from a secreted lactococcal protein, and the sequence has been re‐engineered to enhance secretion levels by increasing the positive charge of the peptide.^[^ ^140^ ^]^ Consequently, the delivery of therapeutics, such as the release of antimicrobial nanobodies to reduce the mortality rate of infected chickens,^[^ ^141^ ^]^ the secretion of factors to assist wound healing,^[^ ^142^ ^]^ and immunomodulatory factors to combat tumors, have been achieved.^[^ ^143^ ^]^

*Escherichia Coli Nissle*. Owing to its extensive use in laboratory settings, *E. coli* is one of the most comprehensively understood microorganisms. The probiotic *E. coli* Nissle 1917 (EcN) is closely related to the lab strain *E. coli* K12, and benefits from the extensive repertoire of genetic manipulation strategies that have been developed,^[^ ^144^ ^]^ although, curiously, the *E. coli* toolkit EcoFlex^[^ ^145^ ^]^ has not been used to engineer EcN, to the best of our knowledge. Due to its easy manipulation, there are an abundance of examples of engineering EcN to deliver therapeutic payloads, and the majority of these transform the microbe with plasmids. Here we will discuss the pros and cons of various genetic engineering strategies, using EcN as a primary example, but the discussion of the advantages and disadvantages will broadly apply to most probiotic bioengineering endeavours.

EcN has been bioengineering to produce a range of protein‐based therapeutics, including nanobodies, enzymes, and factors, among others. Much focus has been on developing inducible promoter systems that prevent the microbe from overexpressing proteins and suffering metabolic burdens until expression is triggered. The T7 RNA polymerase was integrated into EcN genome (EcN::T7) and used to trigger the expression of anti‐inflammatory heme‐based proteins^[^ ^146^ ^]^ and sulfotransferase enzyme in batch reactors.^[^ ^147^ ^]^ Enhanced spatiotemporal control of protein expression was explored by the development of a NIR light‐responsive EcN, which could be orally administered and induced in the gut to alleviate colitis in mice^[^ ^85^ ^]^ and applied in cancer immunotherapies.^[^ ^148^ ^]^ Advanced synthetic and genetic tools have enabled the development of smart microbes with autonomous function, moving away from constitutive or induced expression systems to sensing a target microenvironment. Protein expression can be thermally‐triggered using the temperature‐sensitive promoter pTlpA, which is repressed below 36 °C.^[^ ^149^ ^]^ At higher temperatures in vivo, the repressor disassembles and constitutive expression is commenced. Furthermore, environment‐responsive EcN can be built using a self‐regulating genetic circuit that detects exogenous biomarkers to trigger heterologous protein expression in response.^[^ ^150^ ^]^ The NorR transcription factor changes conformation in the presence of nitric oxide (NO) and binds the pNorV promoter to induce transcription,^[^ ^151^ ^]^ useful when the engineered EcN entered inflamed gut regions where NO concentrations are high. The tool was designed to increase the availability of NorR by designing a bicistronic gene comprised of a therapeutic payload and NorR itself, generating an NO‐dependent positive feedback loop to increase protein expression. Although the system exhibited high levels of background expression in EcN, it did express therapeutic nanobodies in an NO concentration‐dependent manner in vitro.

An important aspect of probiotic engineering is the avoidance of antibiotic use for selection. Many engineering studies have employed plasmids to host the assembled gene circuits, but when the microbial population reproduces and grows, the plasmid may be lost—especially if they incur a metabolic burden. To prevent this, selective markers are required, usually an antibiotic resistance gene and the supply of the appropriate antibiotic to keep the plasmid in the viable population. This process can disrupt the native microbiome when used in vivo, and risks transfer of antibiotic resistance through horizontal gene transfer, so is undesirable for probiotic applications. Alternative selection strategies have been explored, such as the use of essential genes or auxotrophic markers,^[^ ^152^ ^]^ toxin/anti‐toxin systems,^[^ ^153^ ^]^ or direct genome integration of synthetic genes. Genomic integration of synthetic genes can be done in EcN, *L. lactis*,^[^ ^154^ ^]^ lactic acid bacteria,^[^ ^127^ ^]^ and is included in the toolkits for *B. subtilis* ^[^ ^118^ ^]^ and *S. cerevisiae*.^[^ ^109^, ^114^ ^]^

The precise delivery of therapeutics to a target region or tissue of the body will maximize their efficacy and reduce off‐target effects. A mechanism that is difficult to achieve with Gram‐negative species, such as EcN, is the secretion of proteins into their local environments because many export machineries simply secrete proteins into the periplasmic space between their inner and outer membranes.^[^ ^155^ ^]^ Alternative strategies explored with EcN have involved the surface display, such as nanobodies on externally located curli fibres,^[^ ^156^ ^]^ or the design of lyse‐and‐release systems.^[^ ^148a^ ^]^ However, recently, a toolkit specifically designed for EcN protein secretion, PROT_3_EcT (PRObiotic type 3 secretion Ec therapeutic), was developed.^[^ ^157^ ^]^ PROT_3_EcT‐4 was able to colonize the gastrointestinal tract of mice and secrete therapeutic nanobodies for at least 14 days, without disrupting the native microbiome.

### 2.3 Recent Advances in Genetically Engineered PLMs for Therapeutic Applications

**Figure 3:**
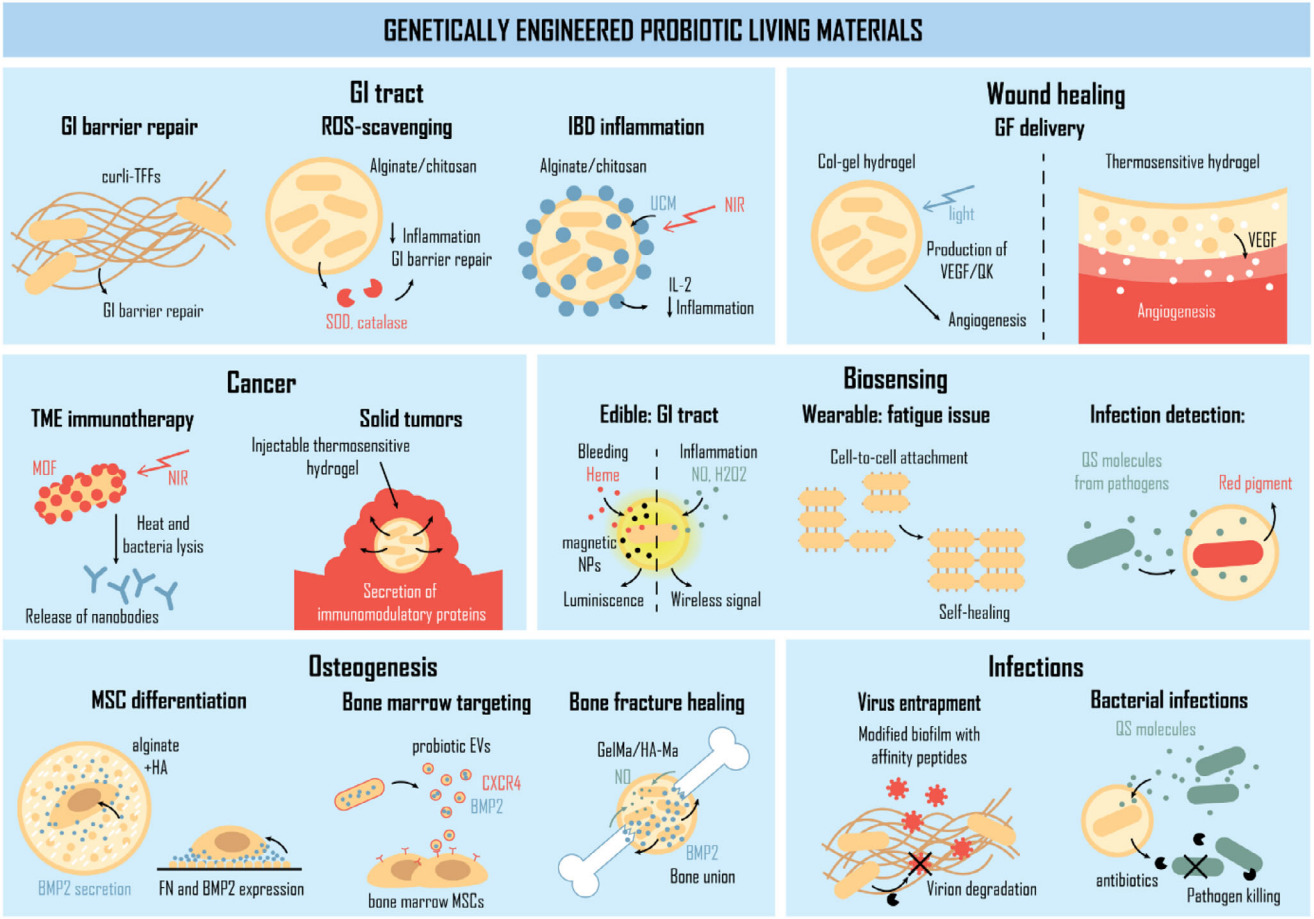
Schematic diagrams of genetically engineered PLMs and their applications. Presently, probiotics are ideal candidates for genetic modification to enhance their current therapeutic effects or to endow them with novel abilities to produce and deliver biotherapeutics at the site of trauma, such as immunomodulatory factors, growth factors, enzymes, or reporter proteins. PTT, photothermal therapy; GI, gastrointestinal; TFFs, trefoil factors, ROS, reactive oxygen species; SOD, superoxide dismutase; IBD, irritable bowel disease; NIR, near infrared light; UCM, up‐conversion material; IL, interleukin; GF, growth factor; VEGF, vascular endothelial growth factor; TME, tumor microenvironment; QK, VEGF‐mimicking peptide; MOF, metal‐organic framework; NO, nitric oxide; H_2_O_2_, hydrogen peroxide; NP, nanoparticle; QS, quorum‐sensing; MSC, mesenchymal stromal cell; HA, hydroxyapatite; BMP2, bone morphogenic protein 2; FN, fibronectin; EV, extracellular vesicle; and CXCR4, C‐X‐C chemokine receptor type 4; GelMa, gelatin methacryloyl; HA‐Ma, hyaluronic acid methacryloyl.

Drugs and biotherapeutics often exhibit greater efficacy when delivered precisely to the site of injury or disease they are intended to treat. However, it can be difficult to direct drugs efficiently, and they then require greater doses to ensure a high enough concentration reaches their target before they are destroyed or metabolized by the body. Living vectors that can home to the disease site, produce and release the therapeutic in situ, can reduce dosages and limit off‐target effects. Engineered probiotics provide the opportunity to design such targeted therapeutics. As discussed in section 2.1, the efficacy of probiotics can be enhanced by combining them with materials platforms, and in section 2.2, they can be genetically engineered to synthesize recombinant therapeutic proteins. Recent approaches to develop next‐generation therapeutics have involved the combination of these strategies to fabricate ELMs containing these recombinant probiotics—probiotic living materials (PLMs; **Figure** **3**)—for diagnostic and therapeutic applications,^[^ ^158^ ^]^ which will be discussed and summarized below (**Table** **2**).

**Table 2:** Genetically engineered Living Probiotic Materials.

Application	Living material	Engineering approach	Non‐living material	Manufacturing technique	Refs.
An edible biosensor to detect bleeding in the upper GI tract	*Escherichia coli* Nissle 1917	Bacteria transformed with a plasmid. Heme‐inducible expression of luxCDABE	Neodynium‐iron‐boron nanoparticles (NdFeB)/ polyvinyl alcohol (PVA) hydrogel	Components mixed in a mold, and PVA gelation triggered. Encapsulation in PVA.	[34]
Edible device for the detection of inflammatory biomarkers (BM)	*Escherichia coli* Nissle 1917	BM inducible recombinase to activate GFP/luxCDABE expression. Plasmid transformation.	Custom microelectronic bioluminescence detector	Bacterial and microelectronics sealed within 3D printed pill capsule (proprietary resin).	[162]
Wearable sensor for muscle and joint monitoring	*Escherichia coli* MG1655	Plasmid transformation. Surface display of adhesive antigen–nanobody pairs to drive bottom‐up self‐assembly.	Proteinaceous matrix	3D‐printing or injection into an elastic thin‐film.	[163]
Biosensor to detect small molecules	*Bacillus subtilis*	Plasmid transformation. Small molecule induces reporter (GFP) expression.	PVA hydrogel	Endospores embedded in PVA hydrogel matrix	[165]
Biosensor to detect pathogen	*Bacillus subtilis*	Plasmid transformation. Small molecule‐induced reporter (RFP) expression.	Alginate–gelatin hydrogel	3D printing	[166]
Living biofilm for disinfecting water	*Escherichia coli*	Self‐assembly with surface‐presented CsgA protein.	Polypropylene	Biofilms grown on polypropylene substrate	[170]
Living biofilm for virus disinfection	*Bacillus subtilis*	Display of virus‐specific affinity peptides and protease expression to destroy viral capsids.	Gauze	Gauze infiltrated with bacteria culture and biofilm grown in situ	[171]
Spores in a hydrogel patch to detect or kill *S. aureus* in wounds	*Bacillus subtilis*	A genomically integrated sensor for *S. aureus* quorum‐sensing molecules	Agarose	3D bioprinting	[172]
Presentation of growth factor bone morphogenic protein 2 (BMP‐2) to stem cells	*Lactococcus lactis*	Inducible expression and secretion of BMP‐2	Alginate and biomineralized hydroxyapatite nanoparticles (HA)	Probiotics and stem cells encapsulated in biomineralized alginate hydrogels	[178]
Presentation of growth factor bone morphogenic protein 2 (BMP‐2) to stem cells	*Lactococcus lactis*	Inducible expression and secretion of BMP‐2	Alginate microgels	Probiotics and stem cells encapsulated in pearl lattice microgels	[179]
Long‐term maintenance of stem cells by providing adhesion motifs	*Lactococcus lactis*	Expression and present FN fragment III_7–10_	Glass coated with poly (ethyl acrylate) (PEA)	Probiotic biofilms grown on modified glass surfaces	[180]
Maintenance of hematopoietic stem cells in vitro	*Lactococcus lactis*	Inducible expression of cytokines	Silanized glass	Probiotic biofilms grown on a modified glass surface	[15a]
Maintenance of hematopoietic stem cells in vitro	*Lactococcus lactis*	Inducible expression of cytokines	PEG hydrogels	Probiotic biofilm grown inside a hydrogel matrix	[181]
Delivery immunomodulatory factors to reduce gut inflammation	*Escherichia coli* Nissle 1917	Inducible expression of IL‐2	Methacrylic acid and ethyl acrylate co‐polymer (Eudragit^®^ L100–55)	Surface coating of probiotic through ionic cross‐linking	[187]
Promote the repair of gut barrier and restore microbiota imbalance	*Escherichia coli* Nissle 1917	Surface presentation of Curli–trefoil factor fusions.	Proteinaceous matrix	Bottom‐up assembly of Curli fibre matrix	[188]
Reduce gut inflammation and restore microbiota imbalance	*Escherichia coli* Nissle 1917	Expression of enzymes to scavenge reactive oxygen species (ROS)	Alginate–chitosan coating	Layer‐by‐layer encapsulation	[81]
Gut–brain axis regulation	*Lactococcus lactis*	Optogenetic expression of gamma‐aminobutyric acid (GAMA), granulocyte‐colony stimulating factor (GCSF), and glucagon‐like peptide‐1 (GLP1)	Alginate–chitosan coating	Layer‐by‐layer encapsulation	[135]
Delivery of immunomodulatory factor IL‐2	*Escherichia coli* Nissle 1917	Optogenetic expression of IL‐2 under control of pDawn	Alginate–chitosan coating and rare‐earth metal‐containing up‐conversion materials (UCMs)	Layer‐by‐layer encapsulation	[85]
Release of immunotherapeutic nanobodies	*Escherichia coli* Nissle 1917	Express anti‐PD‐L1 and anti‐CD9 nanobodies	Zinc metal–organic framework (MOF) loaded with indocyanine green	Surface coating of probiotic	[190]
Immunotherapeutic delivery to solid tumors	*Lactococcus lactis*	Secretion of immunotherapeutic fused to a co‐stimulator	Poloxamer 407	Probiotic encapsulated in poloxamer 407 solution, then gelled upon injection at 37 °C	[191]
Promotion of angiogenesis by delivering vascular endothelial growth factor (VEGF) in wound healing	*Escherichia coli* (*ClearColi*)	Light‐controlled expression and secretion of pro‐angiogenic proteins	Collagen–gelatin (Col–Gel) matrix	Biofilm grown on Col‐Gel film	[193]
Delivery of VEGF in wound healing	*Lactococcus lactis*	Expression and secretion of VEGF	Poloxamer 407 and heparin	Probiotic encapsulated in poloxamer 407 and heparin solution, then gelled upon injection at 37 °C	[194]

#### 2.3.1 Biosensing and Diagnosis

A biosensor comprises a receptor that acts as the sensing element combined with an electronic component that detects and transduces the signal to the processing unit, which generates a signal output.^[^ ^159^ ^]^ They have been widely used in biomedicine to detect pathogens, toxins, and disease biomarkers. Ideally, a biosensor must be non‐invasive, easy to implement, and allow in situ and timely detection of analytes that correlate with biological abnormalities or diseases, enabling low‐cost early diagnosis. In this regard, emerging diagnostic techniques are focusing on the design of ELMs based on microbial sensing circuits. As discussed previously, these circuits simulate the three main modules of a traditional sensor: the genetic input module is the receptor part, the operation module correlates to the electronic transduction system, and the output module with the processing unit. Thus, whole cell‐based biosensors can be combined with different matrices to obtain soft, hard, or flexible miniaturized devices that can be portable, edible, or wearable.^[^ ^160^ ^]^ As an output, these systems produce a reporter molecule, such as a pigment or a fluorescent protein, that can be seen by the naked eye or can be further coupled to electronic detection platforms to quantitatively measure and track the output signal.

Following this rationale, the development of biosensors to detect bleeding in the upper GI tract could avoid the use of invasive endoscopy observation, which is challenging to detect with fecal occult‐blood testing. EcN has been genetically engineered to detect this type of bleeding, which produces luminescence when heme from lysed red blood cells is present.^[^ ^34^, ^161^ ^]^ EcN was embedded into an edible magnetic hydrogel made of PVA and silica‐coated NdFeB (neodymium‐iron‐boron) microparticles, and after its oral intake, a neodymium magnet was kept on the abdomen skin to retain the whole cell biosensor in the intestine. This allowed a prolonged monitoring and the activation of the gene circuit, leading to detectable luminescence activity in the stool when bleeding was elicited by oral indomethacin intake in mice. On the other hand, more sophisticated systems detect inflammatory markers in the GI tract, which can help in the diagnosis of gut‐related conditions such as the inflammatory bowel disease (IBD). Recently, an edible sub‐1.4 cm^3^ capsule based on genetically modified EcN was combined with a photodetector and low‐power readout circuits, allowing the conversion of the luminescence produced by bacteria after sensing transient biomarkers of inflammation into a wireless signal.^[^ ^162^ ^]^ This system can outperform traditional biosensors by sensing in situ labile biomarkers (e.g., NO, hydrogen peroxide, tetrathionate, and thiosulfate) in real time with a portable device, avoiding invasive techniques and bacterial leaking form the device.

In terms of wearable biosensors, one of the main challenges to be addressed is the fatigue issue caused by several cycles of stretching and twisting. Novel approaches, such as cell‐to‐cell attachment by surface modification, can outperform cell growth‐based healing (which can take up to several hours), leading to the recovery of material properties within several minutes. This has been demonstrated by engineering *E. coli* MG1655 to display nanobodies and the matching antigens on the cell surface, leading to gradual aggregation, which endows the ELM with viscoelastic properties similar to those of a hydrogel.^[^ ^163^ ^]^ This wearable device can be assembled within electromyography sensors, allowing the monitoring of muscle functions and facilitating the diagnosis of muscle‐related diseases. This system was also applied to detect strain in joint bending, and its function was retained for significantly longer times compared to traditional stretchable devices. Other studies have overcome the fatigue issue that wearable devices experience using dermal tattoo biosensors, a promising alternative to blood diagnostics.^[^ ^164^ ^]^ This is a proof‐of‐concept study that can potentially be implemented using probiotic strains. The authors conclude that this system could be modified to sense not only different molecular biomarkers, but also other cues such as temperature changes via RNA thermometers.

*B. subtilis* endospores are especially interesting to develop ELM biosensors due to their high stress resistance against harsh conditions (e.g., nutrient starvation, extreme temperatures and pH, and high osmolarity). They can be genetically modified to express GFP when small molecules such as IPTG are present and can be embedded in a PVA casted onto a nonwoven PET mold.^[^ ^165^ ^]^ *B. subtilis* has also been engineered to produce a red pigment after sensing *P. aeruginosa* autoinducers ‐molecules produced by *Pseudomonas* that play a key role in quorum‐sensing and pathogenesis processes.^[^ ^166^ ^]^ The encapsulation of this probiotic within alginate‐gelatin bioinks allows the printing and further crosslinking of the hydrogel structure with calcium chloride. This PLM can sense the presence of *P. aeruginosa* in clinical samples from patients affected by cystic fibrosis.

#### 2.3.2 Infections

Global pandemics caused by pathogenic viruses and microbes highlighted the need to develop effective materials against these threats. Prevention and treatment are the two main approaches to address these threats. Prevention mainly focuses on vaccination and avoiding the initial infection by using physical or chemical methods to capture or destroy the pathogen.^[^ ^167^ ^]^ If prevention fails and infection occurs, an appropriate treatment must be implemented, which often relies on the application of antiseptics, metals, and antibiotics, which are toxic and lead to the development of antimicrobial resistance (AMR).^[^ ^168^ ^]^ AMR encompasses drug resistance in bacteria, fungi, and even viruses such as HIV, tuberculosis, and malaria, and has been declared as one of the top global public health threats by the WHO, contributing to ≈6 million global deaths in 2019.^[^ ^169^ ^]^ Exposure to antibiotics also contributes to local microbiome depleting, posing a risk of reinfection. In both prevention and treatment, microorganisms have been used in combination with several matrices to sense, remove, degrade, and kill pathogens. To obtain these living materials, bottom‐up approaches are an interesting option to create filters capable of removing virions from aqueous solutions. Taking advantage of protein self‐assembled biofilms, *E. coli* and *B. subtilis* have been engineered to display functional domains fused to the biofilm fibers to capture virions in water with an excellent performance. Hence, *E. coli* engineered to express CsgA fused to the influenza virus binding peptide (C5) has the ability to colonize polypropylene, resulting in a film that can disinfect highly contaminated water by binding this virion.^[^ ^170^ ^]^ Moreover, a kill switch was implemented to reduce the risks associated to the environmental release of genetically modified bacteria. Similarly, *B. subtilis* TasA can be fused to the affinity peptide C40 or the peptide SBP1, trapping influenza viruses and SARS‐CoV‐2, respectively.^[^ ^171^ ^]^ This strain was further engineered to secrete the protease AprE to inactivate viruses by degrading the viral capsid proteins. They also included a quorum‐sensing system (ComQXPA) that prevents bacterial overgrowth.

When dealing with bacterial infections, most approaches advocate on‐site delivery of antibiotics or other antimicrobial molecules (such as bacteriocins and enzymes), to avoid the detrimental effect that standard, non‐localized applications have over the host microbiome. Two of the most challenging infections are caused by *P. aeruginosa* and *S. aureus*, which often lead to highly persistent, severe, and recurrent skin infections, most notably when they involve methicillin‐resistant *S. aureus* (MRSA). Hence, some studies have developed PLMs able to sense pathogens and produce antimicrobial compounds in response. This enables a precise in situ treatment that only occurs in the area colonized by the pathogen. In this context, custom‐printed agarose hydrogels containing engineered *B. subtilis* spores have been developed to detect *S. aureus*.^[^ ^172^ ^]^ Interestingly, this PLM can be dehydrated, stored at room temperature, and rehydrated, triggering the spores to germinate and further perform their programmed functions. These germinated cells function as biosensors for the quorum‐sensing molecules of *S. aureus*. They also engineered *B. subtilis* to produce antibiotics, namely lysostaphin or thiocillin, which kill the pathogen but not the probiotic. Drawing on this work, PLMs based on *B. subtilis* can be modulated to target bacterial infections and maintain their function after prolonged storage and exposure to extreme environmental conditions (e.g., ethanol, heat, cold, UV radiation, osmolarity, dehydration). Other study undertook a similar approach in 2022,^[^ ^173^ ^]^ albeit engineering a different commensal skin bacterium, *Staphylococcus epidermidis* ATCC12228. The use of commensal bacteria to treat skin infections would result in a minimal alteration of the microbial ecosystem, as no foreign species are introduced into the target niche. For example, the integration of the sensing circuit in *S. epidermidis* genome via homologous recombination at the *agr* locus allowed it to sense AIP from MRSA strain USA300. Then, these strains were modified to produce different antimicrobial peptides in response to the AIP: LL‐37, elafin, the bacteriocin hiracin, and lysostaphin. Moreover, the incorporation of self‐immunity genes for hiracin and lysostaphin prevented the probiotic self‐killing. While the in vitro performance of this strain was promising, in vivo experiments with germ‐free C57BL6/J mice reflected challenges that probiotic therapies face in real scenario applications: colonization of both *S. aureus* and *S. epidermidis* was highly variable and decreased over time. Moreover, the naïve probiotic strain was also effective at impairing the pathogen growth, suggesting that this strain may not suit mouse skin, and competition for colonizing the skin was taking place.

#### 2.3.3 Osteogenesis

Bone‐related conditions are a major challenge in routine clinical practice. The rapid aging of the populations is accompanied by the progression of osteoporosis and osteoporosis fracture,^[^ ^174^ ^]^ which in addition to other scenarios such as traumatic injuries and surgical removal of tumors, can result in large bone defects and osteomyelitis.^[^ ^175^ ^]^ Indeed, bone is the second most transplanted tissue after blood.^[^ ^176^ ^]^ Hence, there is a growing need for bone regeneration therapies and novel biomaterials for bone grafts. Probiotics are well known to exert immunoregulation and anti‐infection activities, enhancing osseointegration. Recent studies have unlocked new possibilities for the development of smart and dynamic living grafts for bone regeneration using probiotics as biofilms or in combination with bioceramics and hydrogels.^[^ ^177^ ^]^ Furthermore, emerging approaches are implementing genetically engineered probiotics to control or influence human stem cells to move advance dynamic technologies for regenerative medicine. PLMs have been fabricated that bind human mesenchymal stromal cells (MSCs) and promote osteogenic differentiation using genetically modified *L. lactis* that expressed and present cell‐adhesive fibronectin (FN‐III_7–10_) and bone morphogenetic protein 2 (BMP‐2), embedded in a biomineralized alginate matrix^[^ ^178^ ^]^ or pearl lace alginate microgels for low‐cost 3D bioprinting.^[^ ^179^ ^]^ Furthermore, *L. lactis* can be engineered to express FN‐III_7–10_ to fabricate living biointerfaces and hydrogels that enhance MSC adhesion and further differentiation into osteoblasts.^[^ ^180^ ^]^ Recombinant *L. lactis* was also employed to engineer the bone marrow niche by expressing and secreting factors crucial for maintaining hematopoietic stem cell stemness in both 2D living biofilms and 3D polyethylene glycol hydrogels to overcome challenges with expanding these cells in vitro for clinical applications.^[^ ^15^, ^181^ ^]^ On the other hand, EcN has been recently engineered to produce BMP‐2 in response to endogenous NO signals produced by bone fracture injuries. It can be encapsulated in different hydrogels conformations, e.g., microspheres based on gelatin methacryloyl and bulky hyaluronic acid methacryloyl hydrogels, promoting the maturation of the bone callus and supporting bone union in vivo.^[^ ^182^ ^]^

Bacterial derivatives are opening new avenues to address different health conditions, with probiotic EVs being one of the most exploited to maintain the gut‐bone axis homeostasis.^[^ ^183^ ^]^ Recent works employed EVs derived from naïve *Limosilactobacillus reuteri* and *Lactobacillus paracasei* to promote osteogenic differentiation,^[^ ^184^ ^]^ while others focused on the use of EcN that overexpresses BMP‐2 and the C‐X‐C motif chemokine receptor 4 (CXCR4) fused to an EV membrane protein. CXCR4 is a protein that binds the ligand CXC12/SDF1, mainly expressed by bone marrow MSCs, enabling the successful targeting of bone.^[^ ^185^ ^]^

#### 2.3.4 GI Tract

Gastrointestinal disorders are a popular target for genetically engineered PLMs. The gut is an efficient barrier to maintain homeostatic balance. Irritable bowel syndrome (IBS) can derive in inflammatory bowel diseases (IBDs),^[^ ^186^ ^]^ which can inflame the tissue and cause damage to the barrier, and are treated long‐term with drugs or, at worst, complete surgical removal of the bowel. Therefore, advanced strategies to not invasively deliver treatments are desired. In PLMs, the material platform is often designed to protect the microorganism through the digestive tract, and the probiotic is engineered to modulate the immune system and reduce inflammation,^[^ ^85^, ^187^ ^]^ promote tissue repair,^[^ ^188^ ^]^ or restore microbiota imbalances.^[^ ^81^, ^188^ ^]^ Common strategies are to design PLMs to detect, monitor, and/or treat gut‐related problems such as IBD or colitis. For example, PLMs can restore imbalances to the disrupted gut microbiome and reduce inflammation and promote gut repair in IBDs. PLMs exhibiting ROS‐scavenging properties were found to increase the abundance of anti‐inflammatory butyrate‐producing bacteria whilst decreasing the populations of pathogens.^[^ ^81^ ^]^ Furthermore, a living material that could generate its own matrix in a bottom‐up fashion could present anti‐inflammatory trefoil factors (TFFs) on the secreted material to promote intestinal barrier repair.^[^ ^188^ ^]^ The presented TFFs could facilitate integration of the PLM with the mucosal layer of the gut and correlated with the differentiation of T‐helper 17 cells, which regulate the innate and adaptive immune response against extracellular pathogens.

PLMs that can dynamically respond to endogenous or exogenous signals have been designed to secrete neuro‐stimulatory factors to regulate the gut–brain axis^[^ ^135^ ^]^ or deliver immunomodulatory factors to reduce inflammation in IBDs and promote gut repair.^[^ ^85^, ^187^ ^]^ Many studies have employed alginate‐chitosan as coatings that are applied to probiotics in a layer‐by‐layer fashion and held together by electrostatics, but in a low pH environment, the chitosan layers are lost through electrostatic repulsion and the encapsulated microorganisms released.^[^ ^85^, ^135^, ^187^ ^]^ This material has been used in combination with up‐conversion materials (UCM), which convert tissue‐penetrating NIR light to shorter wavelengths (blue) to trigger protein expression in optogenetic systems for spatiotemporal control of therapeutic delivery. A plasmid encoding IL‐2 under the control of light‐reactive promoter pDawn was used to transform EcN co‐encapsulated in an alginate‐chitosan coating with UCMs to deliver IL‐2 to the inflamed gut.^[^ ^85^ ^]^ The structure of the gut epithelium was preserved, and levels of pro‐inflammatory markers like myeloperoxidase were reduced. Similarly, arabinose‐induced production of IL‐2 by EcN restored the immune equilibrium in colitis mouse models.^[^ ^187^ ^]^ The EcN surfaces were ionically cross‐linked to Eudragit^®^ L100‐55, a pH‐responsive copolymer of methacrylic acid and ethyl acrylate, commonly used an enteric coating to protect and deliver drugs. Encapsulating the EcN in the copolymer protected it from pH as low as 1.6, similar to gastric fluids, but upon passage into the intestine, the higher pH ionized and dissolved the Eudragit^®^ L100‐55 to release the probiotic. The delivery of the IL‐10 producing EcN promoted the repair of colitis colon tissues and the upregulation of T‐regulatory cells to regulate the immune response. The secretion of low‐levels (20–40 ng mL^−1^) of IL‐2 by the engineered EcN was sufficient to upregulate T‐regulatory cells to control the immune response, and promoted the repair of colitis colon tissues.

#### 2.3.5 Cancer

Immunotherapy is one of the best strategies to combat tumors, but the immunosuppressive tumor microenvironment limits the infiltration of immune cells, reducing its efficacy. PLMs can be agents of immunostimulatory treatments for cancer, as they can offer long‐term and sustained release of immunomodulatory therapeutic molecules, and they can penetrate deep within the cancerous tissue for release directly into the tumor. Probiotics can be engineered to carry and/or produce immunotherapeutic products,^[^ ^189^ ^]^ but it can be difficult to trigger their release into the tumor. Spatiotemporally controlled release can be achieved by chemically engineering the surface of probiotics with photodynamic nanomaterials. For example, a photothermal zinc‐based MOF loaded with indocyanine green was cross‐linked to modified EcN, and in response to NIR light, the MOF heated up and lysed the EcN for the in situ release of the cytoplasmically expressed nanobodies against immune checkpoint molecules anti‐PD‐1 and anti‐PD‐L1.^[^ ^190^ ^]^ Alternatively, *L. lactis* can secrete heterologously expressed proteins. An PLM was designed using a genetically engineered *L. lactis* expressing an immunomodulatory therapeutic fusion protein comprised of Fms‐like tyrosine kinase 3 ligand and co‐stimulator OX40 ligand. Direct injection into a tumor resulted in retention and long‐term administration of the therapeutic, and significant tumor suppression by expanding dendritic cells and the activation of infiltrating effector T cells.^[^ ^143^ ^]^ The drawback was that direct injection into tumors risks bacteria viability owing to the solid texture of tumors and their high internal pressure. Subsequently, the engineered *L. lactis* were encapsulated in a thermosensitive hydrogel to generate a smart PLM delivery system.^[^ ^191^ ^]^ Made of poloxamer 407, the PLM could be prepared as a liquid at room temperature but experienced a sol‐gel transition to form a hydrogel after injection at 37 °C, where it functioned as a reservoir for slow release of the therapeutic fusion protein. Although encapsulation in a hydrogel reduced drug release by almost 50%, sufficient levels of Flt3l were released to drive the recruitment of immature dendritic cells, while the OX40L stimulated both the expression of more OX40L on CD4^+^T cells in the tumor microenvironment and infiltration of the tumor by T effector cells.

#### 2.3.6 Wound Healing

Genetically engineered probiotics in PLMs can be used to promote and enhance the wound healing process. Wound healing can be stimulated by the application of growth factors, but these are potent and elicit damaging off‐target effects.^[^ ^192^ ^]^ Much attention has been given recently to the localization and controlled release of growth factors (GFs) to assist in tissue regeneration, such as the repair of open wounds. In fact, light‐responsive engineered living materials have been developed to deliver GF peptidomimics, with light‐intensity used to modulate their release at physiologically relevant concentrations of 1–20 nM.^[^ ^193^ ^]^ However, the response was variable, partly attributed to the inability of *E. coli* to secrete recombinant protein, and potentially due to the peptidomimics binding to the encapsulating polymer. As previously discussed, encapsulated probiotics can assist the wound healing process, and many can actively export proteins. Therefore, PLMs offer the opportunity to design materials that produce, secrete, and deliver GFs to induce tissue repair or immunomodulatory factors to reduce inflammation in a controllable manner to limit their off‐target effects and promote healthy wound healing. Bioengineered *L. lactis* was combined with a thermos‐responsive hybrid hydrogel of poloxamer 407 and heparin, which facilitated both application and maintenance of the bacteria population at the wound site, but also sequestered the secreted GFs through heparin interactions to create a store‐and‐release system to promote therapeutic angiogenesis in diabetic wound healing.^[^ ^194^ ^]^ Vascular endothelial growth factor (VEGF) was fused to the Usp45 secretion peptide so *L. lactis* could express and secrete in response to nisin induction. Without heparin, extracellular VEGF concentrations reached 4.5 ng mL^−1^ before being degraded, whereas when heparin was present, the VEGF was protected and reached 10 ng mL^−1^. The PLM increased the viability and mobilization of human umbilical vein endothelial cells (HUVECs) in in vitro scratch assays and promoted their ability to form vessels. Crucially, the innate ability of *L. lactis* to produce lactic acid induced the M2 macrophage phenotype, which secreted additional factors to reduce inflammation and assist the angiogenesis process.

## 3 ELM Safety and Challenges for Therapeutical Applications

ELMs combining synthetic biology and material science breakthroughs are evolving rapidly, finding potential applications in various fields. Special attention has been given to the development of smart, targeted, and sophisticated therapies. Biomaterials based on bacteria and fungi are playing a key role in the design of innovative approaches to biosensing, treatment, and prevention of health imbalances and diseases. As mentioned previously, dysbiosis of the gut microbiota has been linked with numerous health issues, from intestinal conditions to bone alterations and mental health problems. In this regard, the use of native and genetically engineered probiotics can improve the symptoms of most abnormal situations due to their inherent and conferred beneficial effects by restoring the gut microbiota.^[^ ^195^ ^]^ As discussed above, they are attracting great interest to treat infections and gut conditions, for bone regeneration, wound healing, and to develop cancer therapies, among others. However, before reaching clinical applications, they must comply with several regulatory aspects.

In the European Union, the European Food Safety Authority (EFSA) regulates foods, and the European Medicines Agency (EMA) regulates drugs, while in the United States, the Food and Drug Administration (FDA) deals with both categories. In 2012, the FDA published for the first time a guidance that defines “live biotherapeutic products” (LBPs) as “biological products that contains live microorganisms intended to treat, prevent or cure a disease or condition in humans, and is not a vaccine”.^[^ ^196^ ^]^ Similarly, they also defined the subcategory “recombinant LBP”, which refers to genetically modified microorganisms. Afterward, in 2017, the European Directorate for the Quality of Medicines and healthcare officially accepted the term LBP.^[^ ^197^ ^]^ To be registered as a medicine and qualify for the market, LBP quality requirements must comply with the pharmaceutical standards in both the US and Europe.^[^ ^198^ ^]^ As mentioned above, in 2001 probiotics were defined by FAO and WHO experts as “live microorganisms, that when administered in adequate quantities, confer a health benefit on the host”.^[^ ^69^, ^199^ ^]^ This definition may refer to food supplements or to drugs, depending on the targeted population: food supplements are intended to maintain a healthy state in a healthy population, whereas pharmaceuticals are intended to cure or prevent a pathology in unhealthy people. Thus, it may be sensible to categorize therapeutic products comprising probiotics, LBPs or ELMs, differently to each other, rather than treating them all as one group, or they may need to be classified on a case‐by‐case basis. For example, regarding the above definitions, LBPs can be considered a subclass of ELMs, but not every ELM is an LBP, creating a new regulatory framework gap. LBPs usually consist of a single or a combination of microorganisms (naïve or genetically engineered, combined or not with a protective matrix) to deliver a particular therapeutical compound. However, other ELMs can be designed to be more sophisticated and to address more complex challenges; they often include whole cells or cell derivatives and a matrix with advanced functionalities (not only protection), fulfilling broader medical applications compared to LBPs, such as bone grafts, skin substitutes, and eye lenses. Despite promising results arising from world‐wide research efforts, there are still several safety challenges to address regarding i) microbial‐related concerns, ii) delivery format, iii) future trends regarding sustainability, scalability, and manufacturing, and iv) public perspectives.

### 3.1 ELM Development and Challenges

**Figure 4:**
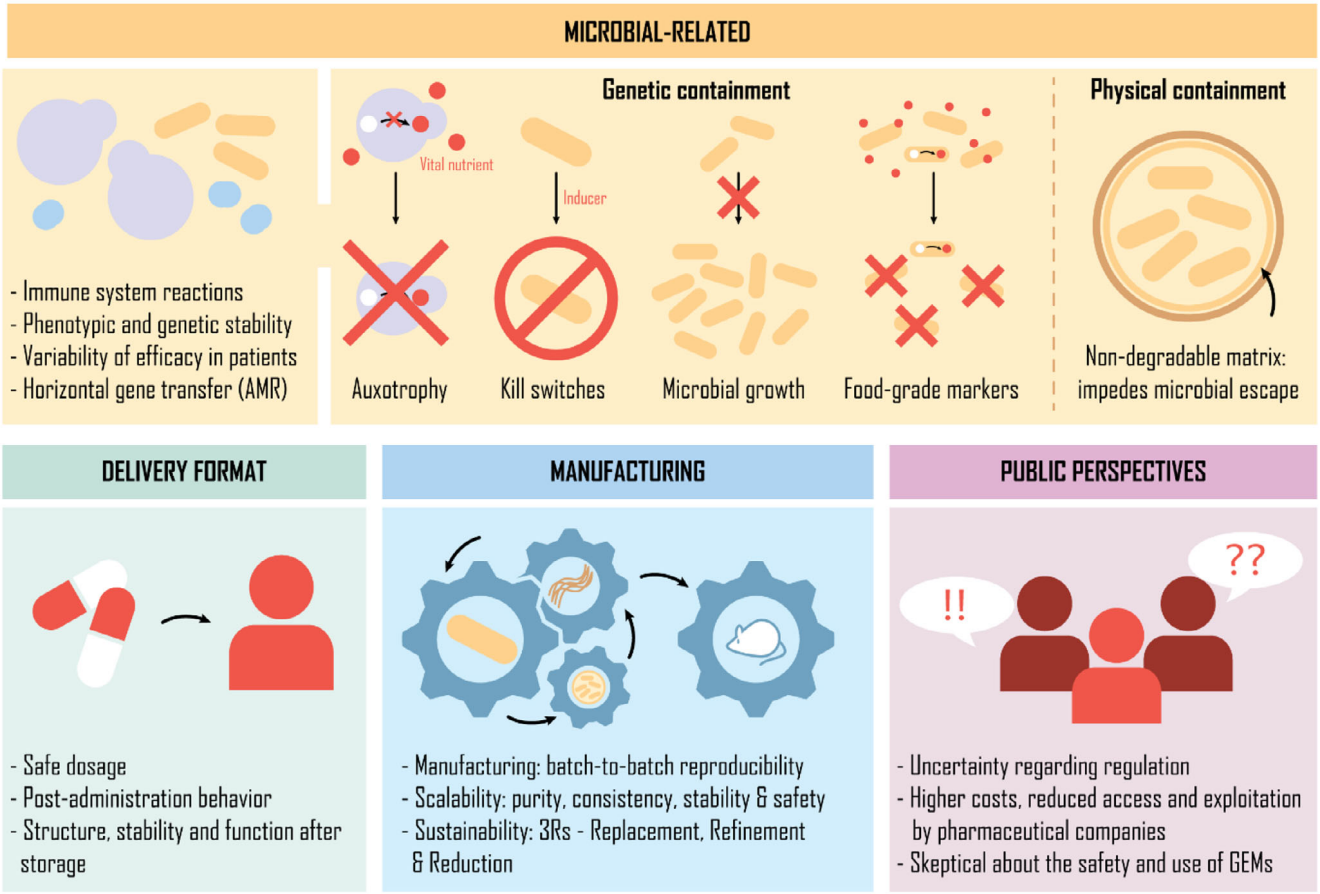
Main challenges and risks that ELM pose for real therapeutic applications related to microbes, delivery format, manufacturing processes, and public perception. Different approaches to avoid the escape of living microbes from the material include genetic and physical containment strategies. AMR, antimicrobial resistance; GEM, Genetically Engineered Microbe.

The development and validation of LBPs and ELMs differs significantly from the tests used to approve traditional drugs: their active compound is usually a living microbe or cellular derivative, which is a dynamic and more complex system. Traditional drugs are often based on a single component—a single molecule or polymer, or combinations thereof—whose safety and quality evaluation guidance is well stablished. To be used as drugs, ELMs must meet these standards, which are more difficult to establish for engineered living entities due to their different and sometimes unpredictable nature. The use of living systems poses several risks at different levels (**Figure** **4**):

*Microbial‐Related Challenges*. The main considerations related to microbes are how the immune system reacts after they have been administered, phenotypic changes of the cells after combination with other materials, their genetic and phenotypic stability during storage and post‐administration, the variability of their efficacy due to patient diversity, and horizontal gene transfer (especially concerning for antibiotic‐based selection markers, which may lead to the spread of AMR). For instance, the prevention of microbial leakage to avoid the release of modified genetic material into the environment can be achieved by implementing physical and genetic containment strategies. Physical containment often requires a non‐degradable matrix that impedes microbial scape, such as crosslinked outer layer hydrogels.^[^ ^200^ ^]^ Alternatively, genetic containment strategies comprise auxotrophy (genes to synthesize an essential nutrient are deleted, such as amino acids, or vitamins, so the microbe depends on exogenous supply and will perish if it escapes the material), inducible genetic kill switches, engineering to control microbial growth, and even the use of food‐grade selection markers (avoids the use of antibiotic resistance genes).^[^ ^201^ ^]^ Risk assessment must be carried out to test the mobility and pathogenicity of the microbes, including metagenome sequencing to thoroughly characterize the strains employed.

*Safe Delivery Format*. Depending on the administration route, a safe dosage and delivery method of the ELM must be determined. Some key points include establishing the quantity of the therapeutic material needed for a particular outcome (which may differ between preclinical experiments and clinical trials), how the biomaterial behaves after administration (e.g., are microbes escaping and reaching the bloodstream causing a potential sepsis, are they boosting an non‐desirable immune reaction, is the material biodegradable and bioresorbable), and the structure, stability and function of the ELM after storage and under different environmental conditions. The translation from in vitro to in vivo experiments and finally humans is a well‐known bottleneck. Even for simple unmodified probiotics, promising in vitro results can be obtained, but in vivo experiments may not provide predictable or consistent outcomes due to variability in data collection methodologies or whether the readouts have any clinical significance,^[^ ^74^ ^]^ and the same can occur when translating in vivo results to humans.

*Sustainability, Scalability, and Manufacturing*. Manufacturing processes rely on the stability and efficacy of the biomaterial, and the reproducibility from batch‐to‐batch production. Optimization of the manufacturing processes must be performed to ensure that the therapeutics are pure, stable, and to assure consistency and safety through quality controls. Optimization could also be achieved at the genetic level. For example, most PLMs function by secreting or releasing a therapeutic product into their surroundings. However, although many studies claim or report secretion from their microbial chassis, the underlying mechanism through which this process occurs is often not described or hypothesized, and the amount of active therapeutic secreted is often not determined and reported. To improve on the design and efficacy of PLMs, engineers should determine secretion efficiencies and report the export mechanism. It is likely that secretion can be enhanced and optimized in many studies by exploiting endogenous machinery by incorporating a secretion tag (if the therapeutic is a protein), or including a characterized secretion system in the chassis, as described in section 2.2. The effect of other materials or scaffolds on the release of the therapeutics should also be considered during design and manufacturing.

Additionally, considering all the ethical concerns that animal use poses, future sustainability perspectives are promoting the 3Rs (Replacement, Refinement, and Reduction) in animal research, with a special emphasis on replacement.^[^ ^202^ ^]^ Thus, potential replacement strategies can employ computational toxicology, artificial intelligence, and machine learning models that can analyze large datasets to predict biological responses,^[^ ^203^ ^]^ or 3D printed tissues and organoids based on human‐derived cells to build healthy or disease models.^[^ ^204^ ^]^

*Public Perspectives*. Although probiotic products are available worldwide, there is uncertainty regarding the regulation of these products. Vulnerable patients express concerns about the regulation of probiotics as therapeutics, which might be accompanied by higher costs, and reduced access, and exploitation by pharmaceutical companies. Also, the general public may be skeptical about the safety and use of genetically engineered probiotics, and they consider that probiotic strains must follow the same regulatory framework as pharmaceutical drugs.^[^ ^205^ ^]^

### 3.2 ELM Regulation, Commercialization, and Clinical Trials

Regarding the steps required to reach the market, there are some important aspects in the design of therapeutics that must be considered. The first step is to consider the scalability of the product and possible interactions with the environment, including both positive and negative reactions. Afterward, following FDA guidance, crucial activities regarding Chemistry, Manufacturing, and Controls must be put in place, which include the assessment of variability across batches, stability in different storage conditions, and shelf‐life of the product, and, in case of new therapeutics like ELMs, any uncertainties related to the manufacturing technique employed.^[^ ^206^ ^]^ In Europe, the guidance on Good Manufacturing Practice (GMP) must be followed to ensure the quality, safety, and efficacy of the product, yet its applicability remains unclear since it does not include the use of living cells.^[^ ^207^ ^]^ GMP guidelines address basic topics, but other critical points remain unclear, such as bioburden detection and biocontainment.^[^ ^208^ ^]^ Thus, the biosafety of the novel biomaterial must be addressed before stepping into clinical trials by conducting pre‐clinical studies. Traditional non‐clinical strategies involve the identification and characterization of the material—sequencing, multi‐omics, chemical and structural analysis—followed by in vitro studies. Such studies are essential to determine the mechanism of action of the ELM at the tissue level, which can also assist in predicting or explaining outcomes in further clinical trials. Some in vitro models, such as organoids and organ‐on‐a‐chip devices,^[^ ^204^ ^]^ can resemble healthy or disease conditions and mimic the physiological environment. However, insights into systemic mechanisms are needed to demonstrate the safety of the biotherapeutic, which relies in the use of animal models. Hence, the use of in vitro models lay the groundwork for optimal in vivo study designs. These in vivo models can be as simple as *Drosophila melanogaster* and *Caenorhabditis elegans*, which are easy to manipulate and involve lower costs, allowing the safety assessment of different materials and as disease models.^[^ ^209^ ^]^ However, the prediction of in vivo responses in humans using these simple models is challenging, and thus mammalian animal models are often preferred, e.g. mice, rabbits, pigs. These models are used to identify immune rejections, tumorigenicity, or any abnormal cell proliferation from both the biomaterial and the host. However, the use of these models raises ethical concerns, and the translation to humans remains challenging, as, for example, and with regards to probiotics, other mammalian hosts possess distinct native microbiomes that may be influenced differently by the treatment. Thus, great efforts are being undertaken to develop more reliable models, trying to make them more ethical and translatable to humans. Once the biosafety, quality, stability and beneficial function of the material has been demonstrated by pre‐clinical tests, clinical trials in humans can be carried out. These studies are divided into three phases: Phase 1 is the safety assessment in a healthy population, Phase 2 is the initial efficacy assessment, Phase 3 is a randomized trial involving larger and different populations to compare the potential product with the standard therapy, and Phase 4 is the post‐marketing surveillance tests after regulatory approval.^[^ ^210^ ^]^

Interestingly, a growing number of non‐model microorganisms are being applied to design LBPs and ELMs. Concerning regulation, both FDA and EMA make a distinction between “chemical entities” and “biotechnological/biological products”, with international drug guidelines clarifying the requirements for marketing authorization: the new product must present a positive benefit‐risk ratio, and be compliant with the quality, biosafety, and efficacy of this legislative framework. However, there is no regulatory pathway in Europe because ELMs do not conform to a separate category. They could fall into the “biotechnological/biological products” group, and may be considered “Medicinal Products”, “Advanced Therapeutic Medicinal Products (ATMPs)”, or combined products. Additionally, ELMs are not considered “medical devices” because the medical device expert panels Regulation (EU) 2017/745 does not apply to them since they contain living cells or cell derivatives.^[^ ^211^ ^]^ ELM classification is especially challenging due to their highly variable nature and applications, which means that the regulatory pathway should be designed depending on the intended use. In view of the need for new verification methods and regulatory pathways for emerging ELMs, the EMA offers scientific advice to support the qualification of innovative development methods for an intended purpose. This advice is given by EMA's Committee for Medicinal Products for Human Use (CHMP) on the basis of recommendations by the Scientific Advice Working Party (SAWP). Actually, EMA recently published an action plan outlining the considerations and steps needed to future‐proof the qualification of novel methodologies, covering actions in 2024 and 2025.^[^ ^212^ ^]^

In the context of commercialization, although complex ELMs have not been approved yet for biomedical applications, some LBPs (based on single genetically engineered cells) are being tested in clinical trials, while others are commercially available,^[^ ^213^ ^]^ summarized in **Table** **3**. Since 2019, two genetically engineered probiotics, from the company ZBiotics, have reached the market: *B. subtilis* ZB423^TM^ expressing levansucrase to make prebiotic fiber, and *B. subtilis* ZB183^TM^ expressing acetaldehyde dehydrogenase to metabolize alcohol and avoid hangovers.^[^ ^214^ ^]^ To prevent any bias in the experimentation and interpretation of data, they contracted Adgyl Lifesciences to subject *B. subtilis* ZB423^TM^ to safety and functionality testing, who demonstrated that the probiotic worked as intended, was completely safe, and FDA‐compliant.^[^ ^215^ ^]^ They also ensure the safety, quality, and efficacy of every batch. Regarding disease treatments, one genetic therapy has been approved (chimeric antigen receptor (CAR) T‐cell therapies), highlighting the challenges awaiting ELM/PLM clinical approval.^[^ ^216^ ^]^ In 2017, the FDA approved for the first time the use of this therapy to treat children with acute lymphoblastic leukemia. Although promising results have been obtained regarding acute cancer cases, CAR T‐cell therapies can cause severe side effects, such as infections and a drop of B‐cell population, highlighting the unpredictable interactions between genetically engineered cells and living organisms.

**Table 3:** LBPs commercially available or under clinical trial testing.

Name	Company	Target	Genetic engineering approach	Clinical trial	Refs.
*B. subtilis* ZB423^TM^	ZBiotics	Help digestion	Expresses levansucrase (prebiotic fiber)	–	[214]
*B. subtilis* ZB183^TM^	ZBiotics	Metabolize alcohol to avoid a hangover	Expresses acetaldehyde dehydrogenase	–	[214]
EcN SYNB1934v1	Synlogic	Phenylketonuria	Uptakes phenylalanine and converts it into non‐toxic metabolites	Phase 3 completed (2024)	NCT05764239
*S. epidermidis* ATR12‐351	Azitra	Netherton syndrome	Expresses a subunit of human lymphoepithelial Kazal‐type‐related inhibitor (LEKTI)	Recruiting for Phase 1 (2025)	NCT06137157
*L. lactis* AG011	ActoGeniX N.V.	Ulcerative colitis	Secretes IL‐10	Phase 2a completed (2009). No significant differences compared to placebo	NCT00729872
*L. lactis* AG013	ActoGeniX N.V.	Oral mucositis in patients receiving chemotherapy	Secretes the Trefoil Factor 1	Phase 1b (2012)	NCT00938080
*Lactococcus cremoris* AUP1602‐C	Aurealis Oy	Diabetic foot ulcer	Secretes human FGF‐2, IL‐4 and CSF‐1	Recruiting for phase 2 (2025)	NCT06111183 EudraCT 2022‐502048‐10‐00
*L. reuteri* ILP100‐Topical	Ilya Pharma	Wound healing in diabetic foot ulcers	Secretes CXCL12	Phase 2 completed (2025)	NCT05608187 EudraCT Number 2021‐000563‐69

All these recent advances using genetically engineered living cells are paving the way for the design of novel regulatory pathways for the legalization of more complex ELMs for therapeutical applications. The use of probiotics in the design of these ELMs is of particular interest in view of the recent introduction of genetically engineered *B. subtilis* into the market by ZBiotics, which sets a precedent in the regulatory approval. However, the biosafety of probiotic strains must be carefully evaluated, since they can pose a risk for compromised human populations. For instance, most ELMs rely on *E. coli* for its ease of use, given the robust and easy‐to‐implement genetic tools available, yet most Gram‐negative strains are well‐known to contain lipopolysaccharides (LPSs) in their cell envelope, which are considered pyrogenic endotoxins in humans, raising safety concerns.^[^ ^217^ ^]^ Among these strains, the probiotic EcN also harbors a pathogenic locus in its genome that produces colibactin, a potential genotoxic metabolite that could play a role in the initiation of colorectal cancer by inducing DNA breaks in host cells.^[^ ^218^ ^]^ On the other hand, although very rare, some pathogenic behaviors of probiotics have been reported in both healthy and diseased populations, and people with weakened immune systems and compromised gut barriers are more prone to develop infections and even sepsis after the administration of probiotics. In this regard, some reports include bacteremia caused by *B. subtilis* in a female with congenital liver fibrosis,^[^ ^219^ ^]^ in a patient with esophageal perforation,^[^ ^220^ ^]^ and even in an immunocompetent male;^[^ ^221^ ^]^ *L. lactis* bacteremia after probiotic supplementation therapy,^[^ ^222^ ^]^ and a case of endocarditis and liver abscess;^[^ ^223^ ^]^ and fungemia after *S. boulardii* administration in hospitalized patients (only in 0.11% of recipients).^[^ ^224^ ^]^ Fortunately, antibiotic treatment was effective in resolving such conditions with no relapses. These cases reinforce the need for a more carefully tailored policy guidance toward the vulnerable population, and a strict monitoring of the patients throughout and after the administration of the treatment.

Currently, only a limited number of probiotics are subjected to extensive genetic engineering strategies and integration with materials to produce PLMs. Lesser‐used probiotics, such as *Bifidobacterium*, *Enterococcus*, or *Streptococcus thermophilus*,^[^ ^225^ ^]^ require the development of genetic tools to better understand their genetics and biochemistries, and to be able to reliably engineer them with predictable properties.^[^ ^226^ ^]^ Additionally, probiotics with unusual biochemistries, such as *Propionibacterium*, which can synthesize propionic acid along with vitamin B12, may be a future chassis for the production of small‐molecule therapeutics.^[^ ^227^ ^]^ Thus, in the coming years, the expansion in the repertoire of engineerable probiotics and standardized genetic tools for more health‐promoting microorganisms is expected, which would be valuable to the field of PLMs. Additionally, it may be advantageous to harness currently non‐probiotic, commensal organisms in living therapeutic design. Commensals do not necessarily provide general health benefits like probiotics, but they are attracting great attention and may be ideal candidates for engineering to confer to the host health‐promoting or therapeutic properties.^[^ ^228^ ^]^ Thinking more into the long‐term future, this can pave the way to more personalized treatments based on the patient microbiome to treat specific conditions, in a similar way to how fecal transplants work. For example, *Bacteroides* spp. is the most abundantly found commensal microbe in the GI tract,^[^ ^229^ ^]^ and has recently been proposed as a potential next‐generation probiotic.^[^ ^230^ ^]^ *Bacterioides* is a well‐understood species^[^ ^231^ ^]^ that has been subject to many genetic engineering efforts. For example, *Bacteroides ovatus* was engineered to express and secrete human growth factors,^[^ ^232^ ^]^ and *Bacteroides thetaiotamicron* has had several genetic editing tools established, including characterized genetic elements,^[^ ^233^ ^]^ promoters regulated by synthetic inducers,^[^ ^234^ ^]^ and an efficient genome editing tool using CRISPR‐Cas9.^[^ ^235^ ^]^ Promisingly, *Bacteroides thetaiotamicron* has been engineered to sense and respond to different biomarkers present in varying concentrations in different regions of the gut, though only expressed a luminescent reporter rather than deliver a therapeutic.^[^ ^236^ ^]^

The human body is host to many microbial communities across many different regions, both internally and externally.^[^ ^237^ ^]^ Many established probiotics are specifically endogenous to certain locations, but, although they are known to provide positive health benefits when delivered, their influence on other local microbiota or tissues may be unknown or unpredictable. Therefore, the design of innovative ELMs/PLMs for deployment in a specific region of the body will potentially focus on the identification of genetically tractable probiotics or commensals endogenous to that tissue or region. For example, *L. plantarum* was recently identified as the most abundant species of the nasal microbiome, and then engineered it to secrete recombinantly expressed hormones. When delivered to the nasal cavity, the factors could bypass the blood–brain barrier by crossing the olfactory epithelium.^[^ ^238^ ^]^ These recent advances are opening new possibilities, and an increasing number of works are focusing on the identification of new microbial chassis isolated from humans, avoiding the use of model probiotics, that may be non‐native to the target tissue.

### 3.3 Emerging Technologies in ELM Design

Emerging technologies are leading to rapid progress in synthetic biology and therapeutic advances. Among them, artificial intelligence (AI) is gaining great attention in the field of healthcare. AI is a technology that enables machines to perform tasks, such as reasoning, problem‐solving, and decision‐making.^[^ ^239^ ^]^ Derivative concepts have emerged from AI in the last 70 years, such as machine learning (ML), deep learning (DL), and generative AI (Gen AI).^[^ ^240^ ^]^

Since AI systems can process large amounts of data and recognize patterns, it opens new avenues in different fields related to biotechnology and healthcare, such as drug discovery, treatment, diagnosis, surgery, and patient monitoring.^[^ ^241^ ^]^ Common advantages that AI offers include the automation of repetitive tasks, the reduction of human errors, more and faster insight from data, and enhanced decision‐making with more accurate predictions. Hence, DL has been widely employed for image‐based diagnosis (using radiology‐based images, to detect skin lesions, ophthalmology conditions, and for histopathological testing),^[^ ^242^ ^]^ and in the field of infectious diseases for drug discovery, infection mechanisms, prevention, and diagnostics.^[^ ^243^ ^]^ For instance, it has been successfully applied to determine positive and negative HIV tests in low‐ and middle‐income countries.^[^ ^244^ ^]^ However, the use of AI comes with a number of challenges: it relies on large high‐quality data for model training; human bias and noise may cause a model to fail when applied in a different environment; the results are sometimes difficult to interpret; collecting, storing, and sharing sensitive data remains a challenge; and clinical diagnostic and treatment must take into account the patient's preferences, values, social environment, and medical history.^[^ ^242^ ^]^ Furthermore, ML models can evolve as more data is collected, which presents a challenge to regulatory agencies, while the legal system must clarify which entity holds the liability, insurance, and coverage in case of negligence if AI has supported a medical decision.

Despite the legal and ethical challenges that AI poses when implemented in healthcare for diagnosis, it arises as a powerful tool in synthetic biology. DL has been widely applied to predict the function and design new sequences and approaches using CRISPR,^[^ ^245^ ^]^ RNA synthetic biology (riboswitches, riboregulators, ribozymes, etc.)^[^ ^246^ ^]^ and toehold switches,^[^ ^247^ ^]^ metabolic engineering to streamline design and improve the production of a metabolite,^[^ ^248^ ^]^ for gene therapy,^[^ ^249^ ^]^ to obtain artificial genetic modules (such as promoters^[^ ^250^ ^]^ and ribosome binding sites^[^ ^251^ ^]^), and in strain design and optimization.^[^ ^252^ ^]^ On the other hand, AI can assist in the development of new materials, functional structures, quantum and life sciences,^[^ ^253^ ^]^ and some of these materials are being extensively studied for biomedical applications.^[^ ^254^ ^]^ AI can accurately predict the drug release profile of a system, taking into account the type, shape, size, and structure of a material, and even the material–drug interactions. Regarding manufacturing techniques, microfluidics and 3D printing have also benefited from the implementation of AI.^[^ ^255^ ^]^ Hence, the use of AI to design new ELMs is only at its infancy, and its use is foreseen to become increasingly widespread in the coming years. In this regard, recent works describe the use of AI in living organoid research.^[^ ^256^ ^]^

Although AI can assist in the design of ELMs, this material must comply with all the legal and safety aspects mentioned above. In this case, AI is a means and not the product. Any novel therapeutic, developed with or without the help of AI, must be approved for pre‐clinical and clinical trials before reaching the market. Thus, regardless of the approach to develop new ELMs or PLMs, there are key translational gaps that remain as great challenges: biological safety, delivery method, reproducibility, scalability, long‐term stability and function, alongside regulatory gaps, ethical concerns, and public perspectives. In the coming years, it is expected that AI‐assisted emerging technologies will speed up commercialization by helping in the selection of the most cost‐effective approach or the best route to reach the market. For instance, AI can highlight which technologies and materials can enter more easily the market and the clinic considering the FDA and EMA guidelines, reducing the costs and the time for clinical trial design and approval.

Although ELMs are approaching clinical relevance, most are still in the prototype and preclinical stage, and routine use in clinics is still years away. However, depending on the use case, some ELMs may reach the market more promptly than others. When it comes to biosensors, especially those that do not require direct contact, it is likely that they can reach the market more easily since they can bypass some biosafety requirements (e.g., systemic and immune system reactions, or safe dosage). Subsequently, the second most likely ELM to be commercialized will be those that have topical applications, like topical biosensors and wound dressing materials (e.g., antimicrobial treatments and dressings for tissue regeneration). There is a real need for topical wound care, with a higher incidence of chronic infections, and at the same time, it is a “low risk” clinical target: it is usually localized and non‐systemic, and easier to monitor and remove if a non‐desired reaction occurs. Moreover, there are some precedents using different dressings (such as bacterial cellulose, e.g., XCell, Biofill, Dermafill)^[^ ^257^ ^]^ already approved by the FDA for topical applications. In the medium to long‐term, GI biosensors and therapeutics may reach the market. Many studies focus on treating GI conditions using probiotics isolated from the gut microbiome, and ingestible materials are exposed to the immune system more gently than if they were implanted or injected. If necessary, the biomaterial can be removed from the GI tract by using invasive techniques such as endoscopy or colonoscopy, but still avoiding a surgical procedure in principle. Finally, any ELM designed to function as an implant (e.g., for bone regeneration) will probably reach the market on a long‐term basis, since it must face strict safety controls and there are no precedents in the surgical implantation of living probiotics in humans.

Hence, future trends in the coming years are looking into more personalized treatments using novel probiotics, commensal bacteria, and the design of robust genetic toolkits, in addition to novel materials. However, there are several challenges to be faced, both at the laboratory and regulatory levels, and new unexpected challenges will arise along the way before its application to the clinic, perhaps in the not‐so‐distant future.

## 4 Conclusion

ELMs are an emerging interdisciplinary class of biomaterials with extensive applications in several fields, such as construction, energy conversion, biosensing, and biomedicine. Microbes are especially relevant in the development of ELMs due to their high growth rate, ability to produce extracellular matrices, and their ease of manipulation. Among them, probiotics that hold GRAS status are of particular relevance in biomedical applications due to their long record of use in humans. Recent development of novel genetic engineering strategies helps in reprogramming probiotics to sense health condition‐related cues (such as disease biomarkers) and perform a localized response, enabling a dynamic, less invasive, and more targeted treatment that aims to avoid off‐target or systemic responses. Among them, EcN, *B. subtilis*, *L. lactis*, and *Saccharomyces* are often preferred for PLMs, as they are well‐established chassis for genetic modification. The development of PLMs after the combination of probiotics with suitable matrices enables the incorporation of new or improved features, mainly longer shelf‐life, tunable mechanical and magnetic properties, self‐healing capabilities, and physical biocontainment, overcoming several limitations of traditional treatments for a wide range of health conditions.

However, the implementation of PLMs in real scenarios needs to overcome a number of challenges regarding biosafety and regulatory frameworks. In general, the administration of microbes in vulnerable populations raises safety concerns, such as unpredictable biological behaviors (e.g., immune responses) that differ from those of healthy populations. On top of that, the use of genetically engineered microorganisms (GEMs) prompts other concerns before and after their administration, such as the horizontal gene transfer to other strains, unexpected cell behaviors, phenotypic changes, genetic stability, and reproducibility from batch‐to‐batch production. These undesirable outcomes also apply to probiotics, and although rare, there have been cases of pathogenic behaviors of probiotics in both healthy and vulnerable patients. Hence, the biosafety, effective dosage, delivery method, biocontainment measures, scalability, long‐term stability, and sustainability of any product containing living cells or cell derivatives must be thoroughly assessed in both healthy and unhealthy populations.

Interestingly, the commercialization of two genetically engineered probiotics has set a precedent for human applications of GEMs. Although they were not intended for therapeutic applications, this event was particularly relevant to set a regulatory framework regarding LBPs and will help in the establishment of new guidelines required for the approval of PLMs and ELMs. Special attention must be drawn to the characteristics and risks that the target population may face, along with those of the strains and components of the material. Thus, new legal requirements must focus on the documentation and demonstration of quality, safety, and efficacy, so that the benefit–risk ratio can be assessed regarding their intended use. Additionally, emerging technologies such as AI are helping in the development of new strains and advanced materials, and can also help in the selection of the best route to reach the market and the clinic. Progress in the identification of novel probiotics and other commensal bacteria, the development of genetic tools, and their combination with suitable matrices are essential for the advancement of safer and more targeted therapies, where the field of ELMs holds vast potential.

## Conflict of Interest

The authors declare no conflict of interest.

## Author Contributions

L.S and M.S‐S. developed the concept of the review that was discussed by all authors to implement the final structure. The initial manuscript was written by L.S and G.J.D. and then edited by M.S‐S. L.S. designed all figures in the manuscript. All authors edited the final submitted version.
